# Increased Dose to Organs in Urinary Tract Associates With Measures of Genitourinary Toxicity in Pooled Voxel-Based Analysis of 3 Randomized Phase III Trials

**DOI:** 10.3389/fonc.2020.01174

**Published:** 2020-07-22

**Authors:** Marco Marcello, James W. Denham, Angel Kennedy, Annette Haworth, Allison Steigler, Peter B. Greer, Lois C. Holloway, Jason A. Dowling, Michael G. Jameson, Dale Roach, David J. Joseph, Sarah L. Gulliford, David P. Dearnaley, Matthew R. Sydes, Emma Hall, Martin A. Ebert

**Affiliations:** ^1^Department of Radiation Oncology, Sir Charles Gairdner Hospital, Nedlands, WA, Australia; ^2^Department of Physics, University of Western Australia, Perth, WA, Australia; ^3^School of Medicine and Public Health, University of Newcastle, Callaghan, NSW, Australia; ^4^School of Physics, University of Sydney, Sydney, NSW, Australia; ^5^Prostate Cancer Trials Group, School of Medicine and Public Health, University of Newcastle, Callaghan, NSW, Australia; ^6^School of Mathematical and Physical Sciences, University of Newcastle, Callaghan, NSW, Australia; ^7^Department of Radiation Oncology, Calvary Mater Newcastle, Waratah, NSW, Australia; ^8^Department of Medical Physics, Liverpool Cancer Centre, Liverpool, NSW, Australia; ^9^South Western Sydney Clinical School, University of New South Wales, Kensington, NSW, Australia; ^10^Centre for Medical Radiation Physics, University of Wollongong, Wollongong, NSW, Australia; ^11^CSIRO, St Lucia, QLD, Australia; ^12^Cancer Research Team, Ingham Institute for Applied Medical Research, Liverpool, NSW, Australia; ^13^School of Surgery, University of Western Australia, Perth, WA, Australia; ^14^5D Clinics, Claremont, WA, Australia; ^15^GenesisCare WA, Wembley, WA, Australia; ^16^Radiotherapy Department, University College London Hospitals NHS Foundation Trust, London, United Kingdom; ^17^Department of Medical Physics and Biomedical Engineering, University College London, London, United Kingdom; ^18^Academic UroOncology Unit, The Institute of Cancer Research and the Royal Marsden NHS Trust, London, United Kingdom; ^19^MRC Clinical Trials Unit, Medical Research Council, London, United Kingdom; ^20^Clinical Trials and Statistics Unit, The Institute of Cancer Research, London, United Kingdom

**Keywords:** external beam radiotherapy, prostate cancer, urinary toxicity, voxel-based analysis, dose-toxicity relationships

## Abstract

**Purpose:** Dose information from organ sub-regions has been shown to be more predictive of genitourinary toxicity than whole organ dose volume histogram information. This study aimed to identify anatomically-localized regions where 3D dose is associated with genitourinary toxicities in healthy tissues throughout the pelvic anatomy.

**Methods and Materials:** Dose distributions for up to 656 patients of the Trans-Tasman Radiation Oncology Group 03.04 RADAR trial were deformably registered onto a single exemplar CT dataset. Voxel- based multiple comparison permutation dose difference testing, Cox regression modeling and LASSO feature selection were used to identify regions where 3D dose-increase was associated with late grade ≥ 2 genitourinary dysuria, incontinence and frequency, and late grade ≥ 1 haematuria. This was externally validated by registering dose distributions from the RT01 (up to *n* = 388) and CHHiP (up to *n* = 247) trials onto the same exemplar and repeating the voxel-based tests on each of these data sets. All three datasets were then combined, and the tests repeated.

**Results:** Voxel-based Cox regression and multiple comparison permutation dose difference testing revealed regions where increased dose was correlated with genitourinary toxicity. Increased dose in the vicinity of the membranous and spongy urethra was associated with dysuria for all datasets. Haematuria was similarly correlated with increased dose at the membranous and spongy urethra, for the RADAR, CHHiP, and combined datasets. Some evidence was found for the association between incontinence and increased dose at the internal and external urethral sphincter for RADAR and the internal sphincter alone for the combined dataset. Incontinence was also strongly correlated with dose from posterior oblique beams. Patients with fields extending inferiorly and posteriorly to the CTV, adjacent to the membranous and spongy urethra, were found to experience increased frequency.

**Conclusions:** Anatomically-localized dose-toxicity relationships were determined for late genitourinary symptoms in the urethra and urinary sphincters. Low-intermediate doses to the extraprostatic urethra were associated with risk of late dysuria and haematuria, while dose to the urinary sphincters was associated with incontinence.

## Introduction

External beam radiotherapy (EBRT) is a prominent treatment option for prostate cancer patients (1), resulting in genitourinary (GU) toxicity with an even higher incidence than rectal toxicity (2). Relationships between treatment and patient specific risk factors, and GU toxicity have been established (3–5). More evidence of GU dose-toxicity relationships is required as more conformal techniques (6, 7) have introduced dose-escalated treatments.

Risk estimation used in establishing dose constraints for healthy organs at risk (OARs) associated with GU toxicity, such as the bladder and urethra, is typically based on considering the planned dose to the whole organ according to dose volume histogram (DVH) or dose surface histogram (DSH) information. This is problematic, however, as it ignores potential spatially varied intra-organ radio-sensitivity. Intuitively, planned dose to symptom related sub-regions (SRSs) of the urethra and bladder has been shown to be more predictive of GU symptoms than information derived from whole-organ DVHs (8). Further understanding of the relationship between dose and urinary toxicity at the voxel level could assist in identifying new SRSs, confirm established SRSs, and help provide these SRS with optimal dose constraints. This would restrict dose to healthy tissues with more spatial specificity, and thus help reduce GU toxicity in patients while maintaining tumor control.

Evidence is accumulating for the establishment of relationships between acute and late GU toxicity and spatial dose variance, particularly within the prostatic urethra (8), at various regions on the surface of the bladder (9, 10), the bladder trigone (11–13), the bladder neck (14) and at subregions within the bladder volume (8). No study to date, however, has performed a voxel-based analysis searching for correlation between dose variation and GU toxicity throughout the entire pelvic anatomy without the assumption that dose-toxicity relationships are limited to within OAR volumes or surfaces. This would enable the identification of dose-toxicity relationships in a broader range of the urinary tract, beyond the prostatic urethra to the membranous and spongy urethra. This extended naïve analysis may also improve understanding of how broader dose patterns, such as those representative of treatment technique (e.g., beam arrangement), relate to toxicity.

In this study, multiple voxel-based statistical methods were employed to investigate the association between 3D planned dose and measures of late GU toxicity in the entire pelvic anatomy. Many shortcomings have typically hindered previous voxel-based analyses (15, 16), including misregistration of planned 3D dose distributions, false positive rates due to the large number of voxels being statistically compared, not using time-to-event data, or not controlling for patient baseline characteristics. This study performed a combination of statistical tests to compensate for these shortcomings. High quality planned dose data from three prospective multi-center prostate radiotherapy clinical trials was utilized in order to assess the consistency of derived associations across cohorts, participating centers, employed radiotherapy techniques and overall treatment approach. “Validation” was defined as applying the same voxel-based tests to datasets from two other trials, with one trial providing a cohort similar to that of the primary dataset and the other substantially different (primarily in terms of treatment technique). This validation determined whether the emergent dose-toxicity patterns within the primary dataset were generalizable to these (similar and different) external datasets. This validation also had an exploratory element, in that it enabled the identification of new emergent patterns in the external datasets regardless of whether they matched the patterns in the primary datasets.

## Methods and Materials

### RADAR Trial

Coordinated by the Trans-Tasman Radiation Oncology Group (TROG), the Randomized Androgen Deprivation and Radiotherapy (RADAR) phase 3 factorial trial (TROG 03.04) compared 6 months of androgen deprivation therapy (ADT) plus radiotherapy with 18 months of ADT with the same radiotherapy, with and without bisphosphonates (17, 18). Accruing a total of 1071 men between October 2003 and August 2007, trial patients had T2 – T4 prostate cancer, undergoing dose-escalated 3D conformal EBRT with prescribed doses of 66, 70 or 74 Gy, or 46 Gy EBRT combined with a brachytherapy boost. Plans could be generated with any preferred combination of 3 or more conformal beams. 3D planned dose distributions with corresponding CT images including delineated CTV, rectum and bladder were collected and utilized as the primary dataset for this study. RADAR was the first TROG trial to incorporate full electronic review of the treatment planning data of accrued patients, facilitated by use of the SWAN system (19). See Table 1 for information on each trial summarized for direct comparison.

**Table 1:** Clinical trials information.

	**RADAR**	**RT01**	**CHHiP**
Full name	Randomized Androgen Deprivation and Radiotherapy (TROG 03.04) Trial (17, 18)	A Randomized Trial of High Dose Therapy in Localized Cancer of the Prostate using Conformal Radiotherapy Techniques (20, 21)	Conventional or Hypofractionated High Dose Intensity Modulated Radiotherapy for Prostate Cancer Trial (22, 23)
Descriptors	• Randomized • Phase 3 • Factorial	• Randomized • Phase 3 • Superiority	• Randomized • Phase 3 • Non-inferiority
Goal	Comparison of 6 months of androgen deprivation therapy (ADT) plus radiotherapy with 18 months of ADT with the same radiotherapy	Comparison of 64 Gy standard-dose and 74 Gy dose-escalated conformal radiotherapy	Comparison of conventional and hypofractionated IMRT
Countries	Australia and New Zealand	United Kingdom, New Zealand, Australia	United Kingdom, New Zealand, Rep. of Ireland, Switzerland
Accrual years	Oct 2003 – Aug 2007	Jan 1998 – Dec 2001	Oct 2002 – Jun 2011
Total accrued patients	1071	843	3216
Date data was frozen	June 2015	Aug 2013	Oct 2017
Patients	Intermediate-risk (T2a) or high-risk (T2b+) prostate cancer	T1b – T3a prostate cancer	T1b – T3a prostate cancer
Radiotherapy type	Dose escalated 3D conformal EBRT	Standard or dose escalated 3D conformal EBRT	Dose escalated IMRT
Prescribed dose groups (dose per fraction)	66 Gy (2 Gy), 70 Gy (2 Gy), 74 Gy (2 Gy)	64 Gy (2 Gy), 74 Gy (2 Gy)	57 Gy (3 Gy), 60 Gy (3 Gy), 74 Gy (2 Gy)
Beam arrangements	Any preferred combination of 3 or more conformal beams	3 or 4 beams (anterior/lateral/posterior) for first 64 Gy, with additional 4 or 6 beam boost to 74 Gy	3 or 4 beams (anterior/lateral/posterior) or 5 beams or more if inverse planning utilized
Electronic review of treatment planning data	Full retrospectve review for all patients (19)	No electronic individual plan review (24)	Full prospective case reviews for the first 2 or 3 patients at each center (25)
Manager	TROG Cancer Research, NSW, Australia	Medical Research Clinical Trials Unit, London, UK	Clinical Trials and Statistics Unit, the Institute of Cancer Research, London, UK
Trial registration number	ISRCTN90298520	ISRCTN47772397	ISRCTN97182923
Ethics approval number	Approved by Hunter New England Human Research Ethics Committee Trial ID 03/06/11/3.02	North Thames Multi-center Research Ethics Committee number MREC/97/2/16	Approved by the London Multi-center Research Ethics Committee number 04/MRE02/10

### RT01 Trial

The RT01 phase 3, international, superiority, randomized controlled trial compared dose-escalated conformal radiotherapy with standard-dose conformal radiotherapy (20, 21). Accruing a total of 843 men between January 1998 and December 2001, patients had confirmed T1b – T3a prostate cancer. The patients underwent 3D conformal EBRT with either a conventional prescribed dose of 64 Gy using prescribed arrangements of either 3 or 4 beams, or the same with an additional 4 or 6 beam boost to 74 Gy. ADT was recommended for 6 months. Similar 3D planned dose distributions, CT and delineation data were collected and utilized as the first external validation dataset of this study. The trial was managed by the Medical Research Council Clinical Trials Unit at University College, London.

### CHHiP Trial

The CHHiP randomized phase 3 non-inferiority trial compared conventional and hypofractionated prostate Intensity Modulated Radiotherapy (IMRT) (22, 23). 3,216 men with T1b–T3a localized prostate cancer were accrued to the trial between October 2002 and June 2011. These underwent IMRT with a conventional prescribed dose of 74 Gy in 2 Gy fractions or hypofractionated courses of 60 Gy or 57 Gy in 3 Gy fractions, all with optional IGRT. ADT was recommended for 6 months, but was optional for patients with low risk disease. Similar 3D planned data was utilized as the second external validation data set for this study. Data was limited to an early cohort of CHHiP patients with processed DICOM information available at the time of acquisition. This trial was managed by the Clinical Trials and Statistics Unit at The Institute of Cancer Research, UK.

### 3D Data Preparation

Three CT image templates were chosen from an independent cohort of 39 prostate EBRT patients (26). Pairwise registrations of CT images within this cohort along with registrations between this cohort and the RADAR CT dataset were used to generate a normalized cross correlation similarity matrix. This matrix was used to perform clustering by affinity propagation to select the single most representative patient CT as an exemplar from the initial cohort. This exemplar was the first registration template (T1). Next, an anti-exemplar, most-different from T1, was chosen as a template on which the impact of registration and reference geometry could be tested (T2). Finally, a similar process was used to select a cropped exemplar, enabling analysis to be restricted to a small region including the prostate and immediate surrounding organs (T3). Dose distributions were then deformed onto these templates through application of deformation vector fields obtained from the image-based registrations above. All registration and dose deformation were performed in 3D. See Appendix Section 2 for images of templates and registration pipelines. The 3D dose distributions from all phases of radiotherapy were summed together according to biologically isoeffective 2 Gy per fraction dose (EQD2) (27), using a spatially invariant alpha/beta ratio of 3, resulting in a single distribution for each patient registered onto each template. The number of voxels and dimensions of the CT image of each registration template and corresponding dose distributions are as follows:

T1: 332 × 249 × 64 voxels
voxel size: 1.17 × 1.17 × 2 mm

T2: 327 × 178 × 76 voxels
voxel size: 1.17 × 1.17 × 2.5 mm

T3: 132 × 130 × 129 voxels
voxel size: 1.24 × 1.24 × 1 mm

Dose distributions used in this analysis were uniformly sampled 1 in 2 voxels for T1 and T2 (due to the large number of total voxels). For T3, every voxel was used.

### Genitourinary Toxicity Endpoints

Four time-to-event GU toxicity endpoints were included for analysis: urinary dysuria, haematuria, incontinence and frequency. For each endpoint, an event consisted of the first peak grade ≥ 2 occurrence during follow-up. For haematuria, however, grade ≥ 1 events were considered instead, due to the rarity of grade ≥ 2 events in the RADAR cohort. All toxicity events were late (> 3 months). All patients who experienced baseline toxicity of grade ≥ 1 were removed from analysis, apart from potential baseline dysuria and haematuria patients from the RT01 dataset, as this information was not available. Physician assisted toxicity grading was performed according to the Late Effects on Normal Tissue, Subjective, Objective, Management, Analytic (LENT/SOMA) questionnaire (28). For RADAR, patients were routinely followed up, post-treatment, approximately every 3 months for 18 months, every 6 months to 5 years, and annually thereafter. RT01 patients were assessed for toxicities at 6, 12, 18, and 24 months after commencing radiotherapy, and annually thereafter. CHHiP patients were assessed for late side-effects beginning 26 weeks after the start of radiotherapy and every 6 months for 2 years, and then annually thereafter.

Note that all voxel-based tests were repeated for all four endpoints, on all three trial datasets (RADAR, RT01 and CHHiP), as well as on a dataset combining patients from all trials (“Combined”). All three registration templates were used for RADAR for exploration of dose-toxicity associations, but only T1 for RT01, CHHiP and Combined for validation. The permutation and uni-voxel tests were performed using MATLAB R2016b and later versions (MathWorks, Natick MA), while the multi-voxel LASSO test was performed on R 3.6.1 (The R Foundation, Vienna). All 3D results were displayed using ITK-SNAP version 3.8.0 (29).

### Voxel-Based Dose Difference Permutation Test

**Figure 1:**
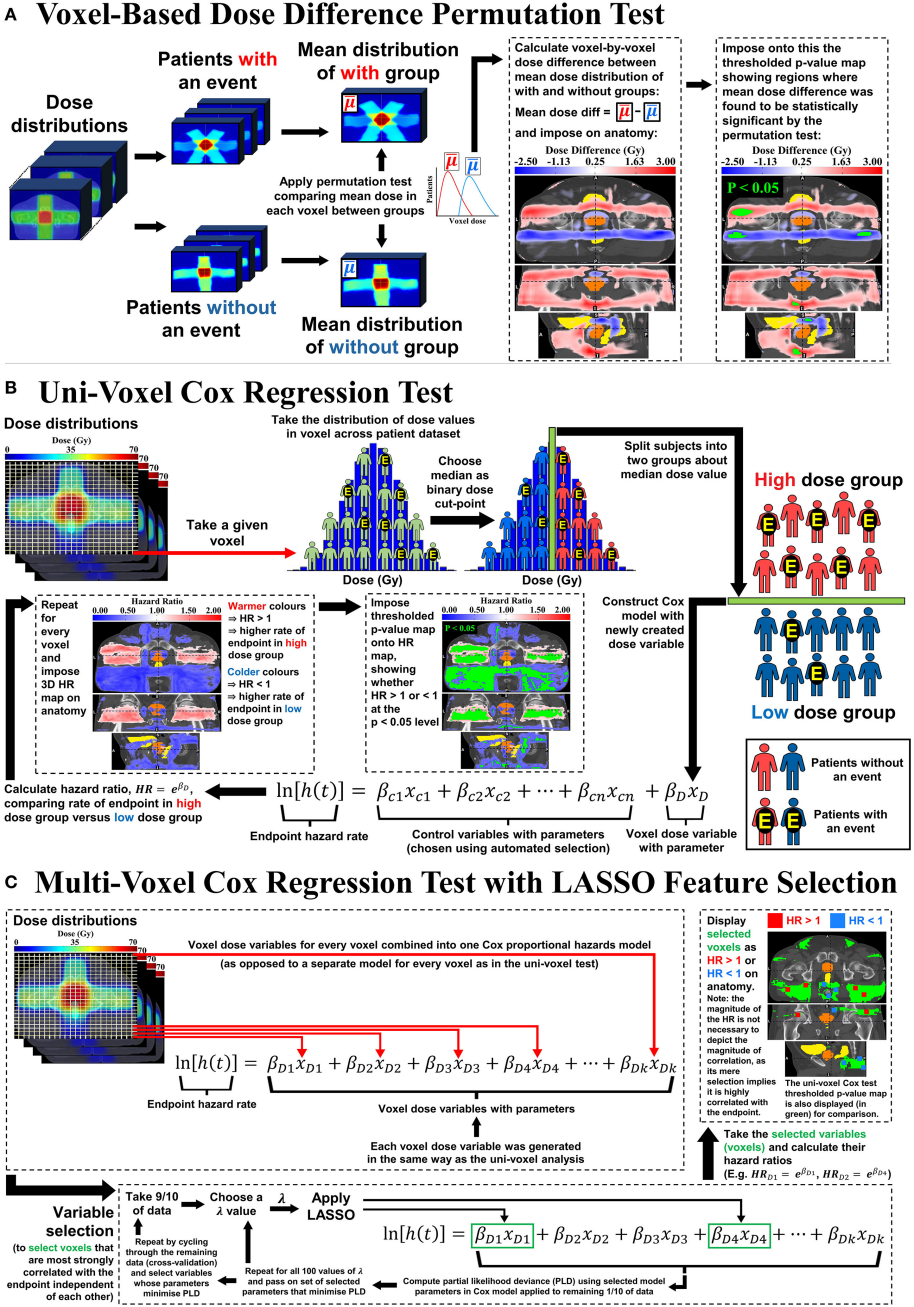
Visual representation of the **(A)** Voxel-Based Dose Difference Permutation Test, **(B)** Uni-Voxel Cox Regression test and **(C)** Multi-Voxel Cox Regression Test with LASSO Feature Selection.

It is recommended that Figure 1 is closely followed while reading through the following descriptions of the voxel-based tests. This test was performed according to the method outlined by Chen et al. (16). Following (Figure 1A), for each given toxicity endpoint, patients were divided according to whether they experienced a toxicity event at any time during follow-up. The mean dose distributions of each group were then compared to each other, voxel-by-voxel, to reveal regions of statistically significant dose difference. This method utilizes a non-parametric permutation-based test in which the group labels (for the with and without toxicity groups) are randomly swapped (permuted) and the dose-comparison repeated for each permutation. 1,000 permutations were performed generating a distribution of test statistics. Each test statistic was calculated as the maximum value across all voxels of the locally normalized dose difference in each voxel for both the true labeling sample and all random permuted samples. The null hypothesis was that the mean of the distribution of dose values in a given voxel for the with toxicity group is not different to the without toxicity group. To find voxels of significant dose-difference between the with and without toxicity groups at any given *p*-value α, a test statistic *T* was calculated as the (1 – α) percentile of the test statistics distribution from the random permuted samples. Voxels where the locally normalized dose difference values for the true labeling sample were greater than *T* are voxels where the dose difference between the with and without toxicity groups is statistically significant at the *p* = α level. In this study, thresholds of *p* < 0.05, *p* < 0.1, *p* < 0.2, and *p* < 0.3 were applied. Multiple *p*-value thresholds were applied in an attempt to thoroughly explore the dose difference, accounting for the conservative nature of the permutation test (see section discussion for further explanation). This test accounts for the multiple statistical testing problem arising from comparing a vast number of voxels (see Appendix A of Chen et al. for more detail). As shown in Figure 1, the mean dose difference map was imposed on the registration template, including the delineated CTV, bladder and rectum. If the dose difference reached statistical significance at one of the given *p*-value thresholds, then the voxels corresponding to this difference (the thresholded *p*-value map) were highlighted in green and imposed onto the dose difference map.

### Uni-Voxel Cox Regression Test

This test generates a separate Cox proportional hazards model for each voxel (hence, “uni”-voxel), testing for association between dose in that voxel and incidence of the toxicity endpoint. Taking a given voxel, patients were divided into two groups about the median of the combined distribution of dose values, as in Figure 1B). The hazard ratio (HR) of the incidence of the endpoint between the high dose value group and low dose value group was then calculated, including a corresponding *p*-value determining whether the HR was significantly greater than or <1 at the *p* < 0.05 level. This HR therefore compares the incidence of toxicity between each dose group, indicating the dose-toxicity relationship at the given voxel. Age, prescribed dose, disease risk, cancer stage, baseline PSA concentration and number of treatment beams were patient baseline characteristics investigated as potential control variables in each model, attempting to eliminate their confounding influence at each voxel (30, 31). These were chosen through an automated selection procedure (see Appendix Section 1 for details). Repeating this entire process for every voxel produced a 3D HR map and corresponding p-value map revealing the relationship between dose and the given toxicity endpoint across the pelvic anatomy. The continuous HR map was first imposed on the anatomical template. Following this, the thresholded p-value map was imposed onto the HR map, showing (in green) voxels where HR < 1 or HR > 1 at the p < 0.05 level.

### Multi-Voxel Cox Regression Test With LASSO Feature Selection

This test is represented in Figure 1C). In contrast to the uni-voxel Cox regression test, this test combined all voxel-dose variables across the pelvic anatomy into a single multivariate Cox regression model (hence, “multi”-voxel). The LASSO [Least Absolute Shrinkage Selection Operator (32)] was then applied to select voxels (voxel-dose variables) that did not correlate with each other in the model, while still correlating strongly with the toxicity endpoint. The LASSO requires a pre-specified variable, λ, that determines the threshold by which features or variables (voxels) in the Cox model are selected. As λ increases, more features are excluded, until none are selected. 100 values of λ were pre-specified, equally spaced from that which selected all voxels to that which selected none. For each value of λ, one-in-ten cross validation was used to test the predictive ability of the resulting Cox model – the model comprised of the voxels selected by the LASSO. The final value of λ was that which maximized the given model's ability to predict the toxicity endpoint hazard rate by minimizing the partial likelihood deviance. The selected voxels were then imposed on the anatomical template, indicating whether HR > 1 or HR < 1 in each case. As with the univoxel Cox regression test, HRs in this test compared the incidence of the endpoint (e.g., dysuria) between the high dose group and low dose group at a given voxel, with the cut-point for dose determined in the same way. The LASSO enabled selection of voxels strongly correlated with the endpoint while accounting for inter-voxel dose correlation and the multiple testing problem.

## Results

### Trial Datasets

Tables 2, 3 show the number of patients from each trial included for each endpoint's respective analysis, with corresponding patient variable and endpoint follow-up information, after patients were excluded due to loss of follow-up, missing data, and considering only patients receiving EBRT alone.

**Table 2:** The number of patients in each trial dataset, broken down by endpoint and baseline variables, including follow-up information, for dysuria and haematuria.

**RADAR**				**RT01**			**CHHiP**			**COMBINED**		
		**Dysuria (grade ≥ 2)**	**Haematuria (grade ≥ 1)**		**Dysuria (grade ≥ 2)**	**Haematuria (grade ≥ 1)**		**Dysuria (grade ≥ 2)**	**Haematuria (grade ≥ 1)**		**Dysuria (grade ≥ 2)**	**Haematuria (grade ≥ 1)**
Total number of patients		595	619	Total number of patients	388	388	Total number of patients	242	247	Total number of patients	1225	1254
Events		79 (13.3%)	86 (13.9%)	Events	36 (9.3%)	52 (13.4%)	Events	11 (4.5%)	21 (8.5%)	Events	126 (10.3%)	159 (12.7%)
Follow-up in months (min, max, med, IQR)		(12, 84, 54, 30)	(5, 95, 53, 30)	Follow-up in months (min, max, med, IQR)	(6, 158, 105, 57)	(6, 158, 102, 55)	Follow-up in months (min, max, med, IQR)	(6, 68, 60, 2)	(6, 68, 60, 2)	Follow-up in months (min, max, med, IQR)	(6, 158, 60, 30)	(5, 158, 61, 30)
Variables	Definitions			Definitions			Definitions			Definitions		
Age^1^	Median	69.4 yrs	69.4 yrs	Median	67.9 yrs	67.9 yrs	Median	67.4 yrs	67.4 yrs	Median	68.4 yrs	68.4 yrs
Prescribed dose	[66 Gy] [70 Gy] [74 Gy]	78 328 189	81 343 195	[64 Gy] [74 Gy]	204 184	204 184	[57 Gy] [60 Gy] [74 Gy]	82 82 78	87 83 77	[66 Gy (RADAR), 64 Gy (RT01), 57 Gy and 60 Gy (CHHiP)] [70 Gy and 74 Gy (RADAR), 74 Gy (RT01), 74 Gy (CHHiP)]	467 857	455 799
Disease risk	[GS ≤ 7] [GS > 7]	418 177	436 183	[T1b/c or T2a with (PSA + (GS - 6)*10) < 15] [T1b/c or T2a with (PSA + (GS - 6)*10) ≥ 15 or T2b/T3a]	110 278	110 278	[T1b/c or T2a with PSA ≤ 10 and GS ≤ 6] [Any of the following: Stage ≥ T2b, 10 < PSA ≤ 20, GS > 6]	57 185	59 188	[Lower risk group patients from each respective dataset] [Higher risk group patients from each respective dataset]	648 676	605 649
Cancer stage	[T2] [T3/T4]	427 168	448 171	[≤ T2a (T1b, T1c, T2a)] [> T2a (T2b, T3a)]	235 153	235 153	[≤ T2a (T1a, T1b, T1c, T2a)] [> T2a (T2b, T2c, T3a)]	177 65	179 68	[Lower cancer stage group patients from each respective dataset] [Higher cancer stage group patients from each respective dataset]	839 386	862 392
Baseline PSA^a^	Median	14.04 ng/ml	14.00 ng/ml	Median	13.80 ng/ml	13.80 ng/ml	Median	11.70 ng/ml	11.70 ng/ml	Median	13.60 ng/ml	13.50 ng/ml
Number of beams	[3 beams] [4 beams] [5 beams] [6 beams] [≥ 7 beams]	65 311 79 84 56	65 331 81 87 55	[3 beams for phase 1 of treament] [4 beams for phase 1 of treament]	228 160	228 160	[≤ 4 beams] [> 4 beams]	212 30	217 35	[≤ 4 beams (RADAR), 3 beams (RT01), ≤ 4 beams (CHHiP)] [> 4 beams (RADAR), 4 beams (RT01), > 4 beams (CHHiP)]	816 409	841 413

a *This variable was divided into two approximately equal subgroups split about the median value*.

**Table 3:** The number of patients in each trial dataset, broken down by endpoint and baseline variables, including follow-up information, for incontinence and frequency.

**RADAR**				**RT01**			**CHHiP**			**COMBINED**		
		**Incontinence (grade ≥ 2)**	**Frequency (grade ≥ 2)**		**Incontinence (grade ≥ 2)**	**Frequency (grade ≥ 2)**		**Incontinence (grade ≥ 2)**	**Frequency (grade ≥ 2)**		**Incontinence (grade ≥ 2)**	**Frequency (grade ≥ 2)**
Total number of patients		647	416	Total number of patients	354	264	Total number of patients	242	206	Total number of patients	1243	886
Events		24 (3.7%)	125 (30.0%)	Events	26 (7.3%)	131 (49.6%)	Events	6 (2.5%)	33 (16.0%)	Events	56 (4.5%)	289 (32.6%)
Follow-up in months (min, max, med, IQR)		(12, 84, 54, 36)	(12, 84, 18, 36)	Follow-up in months (min, max, med, IQR)	(6, 152, 103, 48)	(12, 158, 60, 90)	Follow-up in months (min, max, med, IQR)	(6, 68, 60, 2)	(6, 67, 60, 3)	Follow-up in months (min, max, med, IQR)	(6, 158, 60, 30)	(6, 158, 48, 46)
Variables	Definitions			Definitions			Definitions			Definitions		
Age^a^	Median	69.5 yrs	69.1 yrs	Median	67.6 yrs	68.6 yrs	Median	67.3 yrs	67.3 yrs	Median	68.4 yrs	68.4 yrs
Prescribed dose	[66 Gy] [70 Gy] [74 Gy]	82 358 207	43 230 143	[64 Gy] [74 Gy]	186 168	141 123	[57 Gy] [60 Gy] [74 Gy]	84 81 77	72 70 64	[66 Gy (RADAR), 64 Gy (RT01), 57 Gy and 60 Gy (CHHiP)] [70 Gy and 74 Gy (RADAR), 74 Gy (RT01), 74 Gy (CHHiP)]	433 810	326 560
Disease risk	[GS ≤ 7] [GS > 7]	457 190	288 128	[T1b/c or T2a with (PSA + (GS - 6)*10) < 15] [T1b/c or T2a with (PSA + (GS - 6)*10) ≥ 15 or T2b/T3a]	101 253	74 190	[T1b/c or T2a with PSA ≤ 10 and GS ≤ 6] [Any of the following: Stage ≥ T2b, 10 < PSA ≤ 20, GS > 6]	58 184	51 155	[Lower risk group patients from each respective dataset] [Higher risk group patients from each respective dataset]	616 627	413 473
Cancer stage	[T2] [T3/T4]	465 182	305 111	[≤ T2a (T1b, T1c, T2a)] [> T2a (T2b, T3a)]	216 138	156 108	[≤ T2a (T1a, T1b, T1c, T2a)] [> T2a (T2b, T2c, T3a)]	176 66	152 53	[Lower cancer stage group patients from each respective dataset] [Higher cancer stage group patients from each respective dataset]	857 386	613 273
Baseline PSA^a^	Median	14.04 ng/ml	14.25 ng/ml	Median	13.40 ng/ml	14.00 ng/ml	Median	11.70 ng/ml	11.85 ng/ml	Median	13.45 ng/ml	13.60 ng/ml
Number of beams	[3 beams] [4 beams] [5 beams] [6 beams] [≥ 7 beams]	69 345 85 89 59	44 223 58 52 39	[3 beams for phase 1 of treament] [4 beams for phase 1 of treament]	212 142	159 105	[≤ 4 beams] [> 4 beams]	212 30	181 25	[≤ 4 beams (RADAR), 3 beams (RT01), ≤ 4 beams (CHHiP)] [> 4 beams (RADAR), 4 beams (RT01), > 4 beams (CHHiP)]	838 405	607 279

a *This variable was divided into two approximately equal subgroups split about the median value*.

### Voxel-Based Tests Results

**Figure 2:**
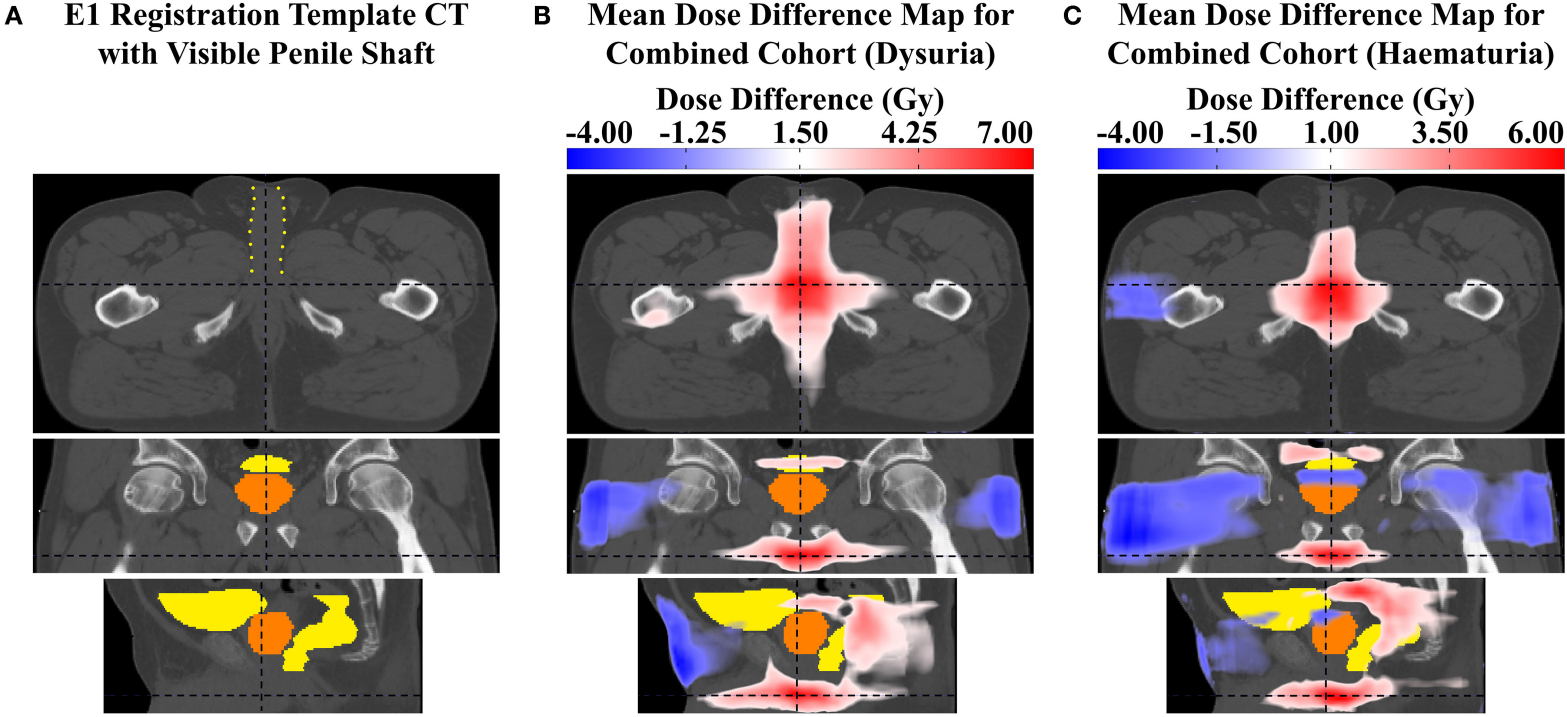
**(A)** slices of the T1 registration template CT image with the penile shaft (containing the spongy urethra) visible despite not being delineated, outlined in yellow dots. Mean dose difference maps between patients with and without **(B)** grade ≥ 2 dysuria and **(C)** grade ≥ 1 haematuria, on approximately the same urethral slice as the image in **(A)** for comparison. It is evident that maximum dose difference is most likely occurring at the urethra.

The tests identified voxel clusters (VCs) and individual voxels within the pelvic anatomy where increased dose was associated with the four genitourinary toxicity endpoints. Several anatomical landmarks (different urethral regions, sphincters etc.) are mentioned in the following descriptions. These structures are not directly visible on the registration template CT images. Their locations are assumed based on their anatomical proximity to (or within) the prostate and the penile shaft, both visible on the registration template CT images. I.e., it is assumed the spongy urethra runs along the central axis of the visible penile shaft (extending approximately 10.2 cm anteriorly from the base of the penile shaft toward the surface of the patient on the T1 template), the membranous urethra is located between the apex of the prostate and the base of the penile shaft (extending approximately 3.4 cm inferiorly from the prostatic apex to the base of the penile shaft on the T1 template), the bladder neck is located where the bladder and prostate delineations meet (near the superior prostate boundary), and the external and internal sphincters are located immediately inferior to the prostatic apex and immediately superior to the central superior boundary of the prostate. Figure 2 shows the visibility of the penile shaft on the T1 registration template CT image. It must be noted that these structures have not been delineated and therefore references to their location are approximate.

The following dose-toxicity patterns from all RADAR datasets on T1 were generally reproduced on the other registration templates (T2 and T3). The patterns were distorted according to the anatomical difference between the templates, but otherwise were similar, suggesting the revealed dose-toxicity association patterns are largely independent of choice of registration template (see Appendix Section 3 for these results).

### Dysuria

**Figure 3:**
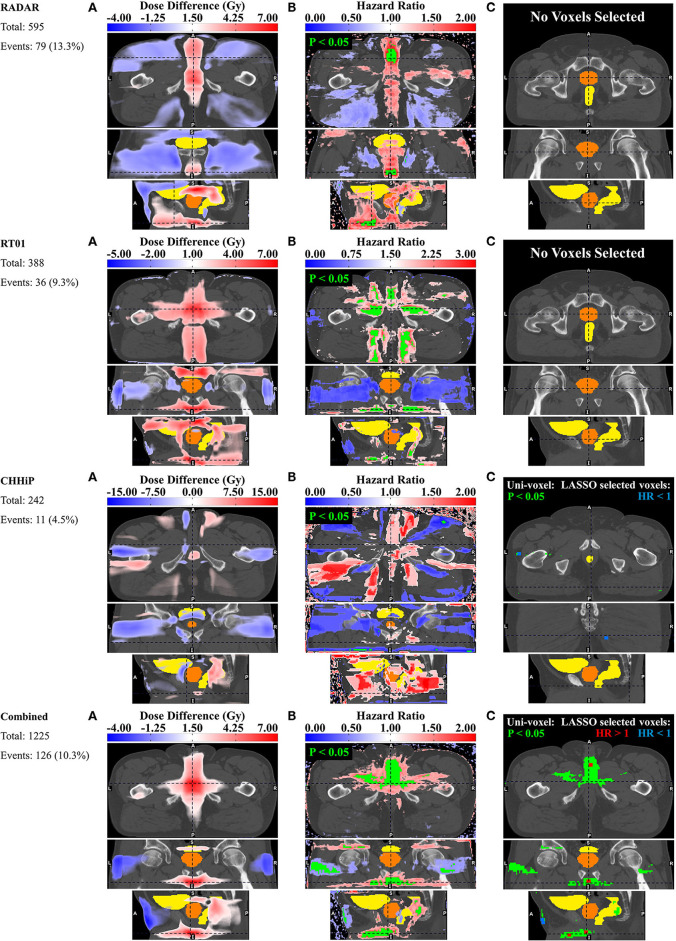
Results for dysuria. Corresponding axial, coronal and sagittal slices (top to bottom) of **(A)** mean dose difference maps, **(B)** uni-voxel Cox regression HR and *p*-value maps and **(C)** multi-voxel Cox regression LASSO HR maps (with uni-voxel *p*-values for comparison), for respective data sets. “No Voxels Selected” implies the LASSO selected no voxels of significant correlation with the endpoint within the patient region. The slices chosen for display are those which coincide with the most dominant emergent dose-endpoint patterns, indicated in corresponding planes with dashed lines. Tones of red correspond to regions where increased dose is associated with incidence of dysuria (HR > 1), while tones of blues correspond to regions where reduced dose is associated with incidence of dysuria (HR < 1). The CTV is delineated in orange while the bladder and rectum are delineated in yellow. Anatomical directions left (L), right (R), superior (S), inferior (I), anterior (A), and posterior (P) are also indicated.

The pelvic dose associations for dysuria are shown in Figure 3. The consistent pattern is an association between a higher incidence of dysuria and increased dose in the membranous urethra and spongy urethra. This is particularly evident in the uni-voxel HR maps, revealing VCs with HR > 1 (*p* < 0.05) in the spongy urethra for RADAR, RT01 and Combined, and VCs with HR > 1 (*p* > 0.05) here for CHHiP. These are most evident in the axial and sagittal planes. For Combined, VCs with HR > 1 (*p* < 0.05) are also present in the vicinity of the membranous urethra. For RT01, HR > 1 (*p* < 0.05) VCs are also found surrounding the extraprostatic urethra, particularly laterally and in the posterior beam region adjacent to the extraprostatic urethra (seen in the axial plane). Although the permutation test found no significant mean dose difference up to the *p* < 0.3 level, the corresponding mean dose difference maps are generally consistent with these associations. Patients who experienced dysuria had up to 7 Gy more planned dose on average in the corresponding associated regions for RADAR, RT01 and Combined, and 4 Gy for CHHiP. **Figure 7** shows that patients with and without dysuria in the combined dataset had total doses of 48.2 and 42.2 Gy, respectively at a point near the membranous urethra, and 19.7 and 16.2 Gy at a point near the spongy urethra. It is also noteworthy that patients experiencing dysuria had up to 7 Gy more dose near the bladder neck region for RADAR and RT01, with patients in each cohort having mean total doses here of approximately 44 and 51 Gy, respectively (see Appendix Section 4 for mean dose distributions). For Combined, the dominant spongy and membranous urethral dose-association is confirmed by the corresponding multi-voxel results, as the LASSO selected voxels with HR > 1 in the same regions as the HR > 1 (p < 0.05) VCs found in the uni-voxel map. The RADAR uni-voxel maps on the T3 template (see Appendix Section 3) confirm the membranous and spongy urethra correlation and reveal some correlated voxels in the prostatic urethra also. In conclusion, patients with increased dose in the vicinity of the membranous and spongy urethra experienced a higher incidence of late grade ≥ 2 dysuria.

### Haematuria

**Figure 4:**
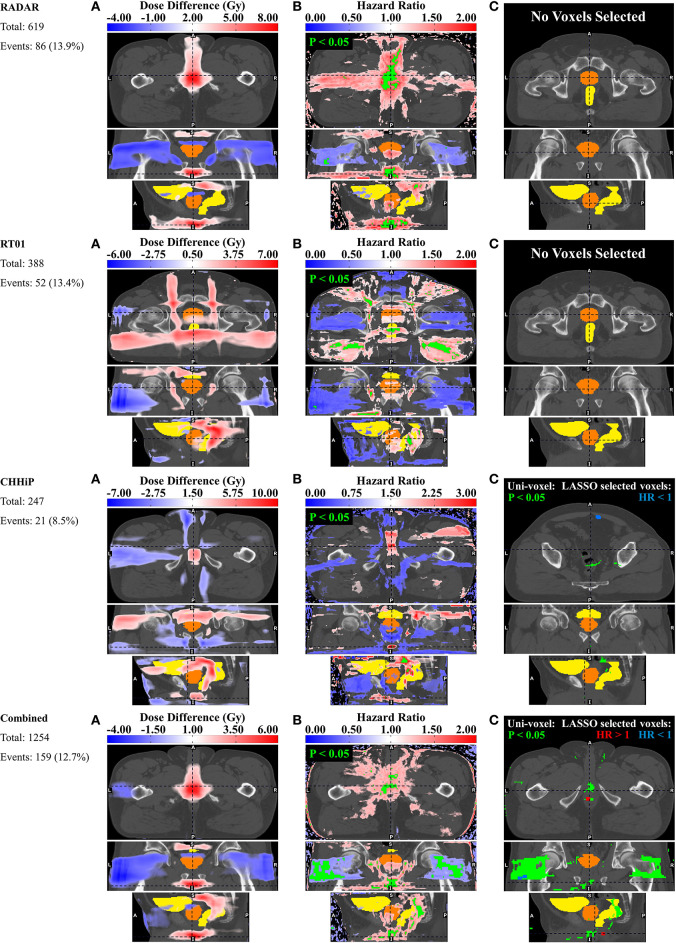
Results for haematuria. Corresponding axial, coronal and sagittal slices (top to bottom) of **(A)** mean dose difference maps, **(B)** uni-voxel Cox regression HR and *p*-value maps and **(C)** multi-voxel Cox regression LASSO HR maps (with uni-voxel *p*-values for comparison), for respective data sets. “No Voxels Selected” implies the LASSO selected no voxels of significant correlation with the endpoint within the patient region. The slices chosen for display are those which coincide with the most dominant emergent dose-endpoint patterns, indicated in corresponding planes with dashed lines. Tones of red correspond to regions where increased dose is associated with incidence of haematuria (HR > 1), while tones of blues correspond to regions where reduced dose is associated with incidence of haematuria (HR < 1). The CTV is delineated in orange while the bladder and rectum are delineated in yellow. Anatomical directions left (L), right (R), superior (S), inferior (I), anterior (A), and posterior (P) are also indicated.

Figure 4 shows the results for haematuria. Similar to dysuria, the major association is between increased dose in the membranous and spongy urethra and increased haematuria. The uni-voxel HR maps show VCs with HR > 1 (*p* < 0.05) in these regions for RADAR (including for T3), CHHiP and Combined. RT01 results are not consistent with these findings, revealing VCs with HR > 1 (*p* < 0.05) in the posterior oblique beam regions. Reduced dose in the lateral beam regions is also correlated with increased haematuria, evident in the HR > 1 (*p* < 0.05) VCs found in these regions for RADAR and Combined, particularly visible on the coronal planes. Although the permutation test found no regions of significant dose difference, the corresponding mean dose difference maps confirm these dominant associations for all datasets. For example, these reveal that patients who experienced haematuria had up to 8, 5, and 6 Gy more dose on average in the vicinity of the membranous and spongy urethra for RADAR, CHHiP and Combined respectively. **Figure 7** shows that patients with and without haematuria in the combined dataset had total doses of 47.4 and 42.3 Gy, respectively at a point near the membranous urethra, and 18.6 and 16.5 Gy at a point near the spongy urethra. The dose difference maps also show that patients with haematuria had up to 5, 8 and 4 Gy more dose on average near the bladder neck/trigone region for RT01, CHHiP and Combined, respectively. The LASSO selected a voxel with HR > 1 directly posterior to the spongy/membranous urethra region for Combined. It also selected HR <1 voxels in the lateral beam region. In conclusion, patients with increased dose in the vicinity of the spongy and membranous urethra experienced a higher incidence of late grade ≥ 1 haematuria. Figure 2 shows how the maximum dose difference for both dysuria and haematuria was located in the extraprostatic urethra (using the visibility of the penile shaft).

### Incontinence

**Figure 5:**
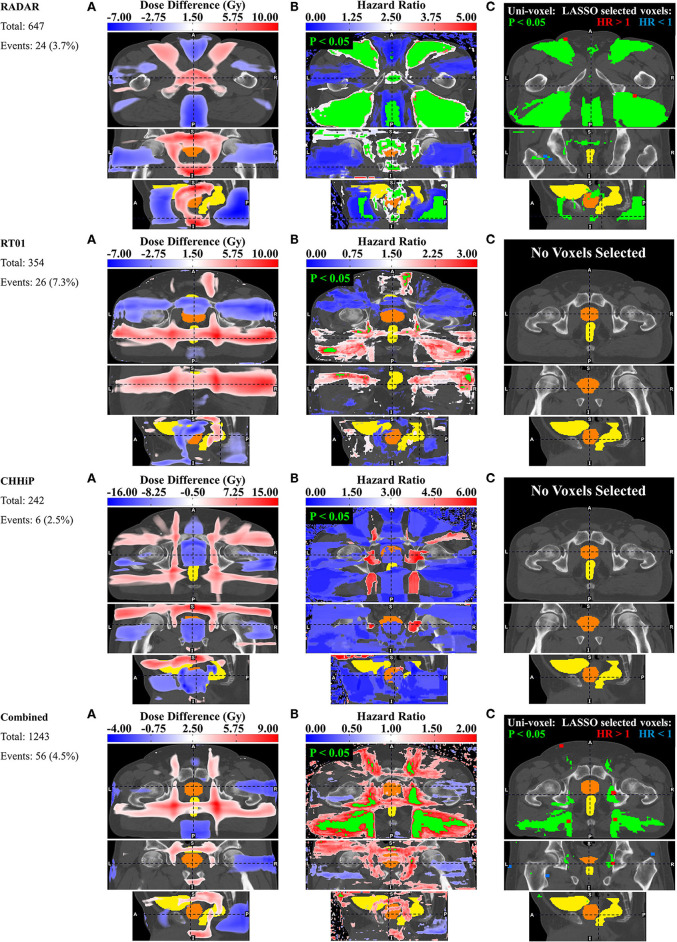
Results for incontinence. Corresponding axial, coronal and sagittal slices (top to bottom) of **(A)** mean dose difference maps, **(B)** uni-voxel Cox regression HR and *p*-value maps and **(C)** multi-voxel Cox regression LASSO HR maps (with uni-voxel *p*-values for comparison), for respective data sets. “No Voxels Selected” implies the LASSO selected no voxels of significant correlation with the endpoint within the patient region. The slices chosen for display are those which coincide with the most dominant emergent dose-endpoint patterns, indicated in corresponding planes with dashed lines. Tones of red correspond to regions where increased dose is associated with incidence of incontinence (HR > 1), while tones of blues correspond to regions where reduced dose is associated with incidence of incontinence (HR < 1). The CTV is delineated in orange while the bladder and rectum are delineated in yellow. Anatomical directions left (L), right (R), superior (S), inferior (I), anterior (A), and posterior (P) are also indicated.

Figure 5 shows the results for incontinence. There is some evidence of an association between incontinence and increased dose at the urethral sphincters, while the dominant association is between incontinence and patients treated with posterior oblique beams and posteriorly extended lateral beams. The uni-voxel HR maps show VCs with HR > 1 (*p* < 0.05) in the vicinity of the internal and external urethral sphincters for RADAR, while a smaller HR > 1 (*p* < 0.05) VC is present near the internal sphincter for Combined, most clearly visible in the coronal plane. Larger VCs with HR > 1 (*p* < 0.05) are present in the vicinity of the internal and external sphincters for RADAR on the T3 template, with the LASSO confirming this internal sphincter association by selecting HR > 1 voxels near the internal sphincter. The permutation test found no VCs of dose difference up to *p* < 0.3, but the corresponding dose difference maps did show that patients with incontinence had up to 10 Gy more dose on average than patients without incontinence near both sphincters for RADAR (more clearly seen on the T3 template), and up to 5 Gy more for Combined. RADAR patients had a mean total dose of ~69 Gy near the internal sphincter and 65 Gy at the external sphincter, reading off the mean dose distribution on the T3 template in Appendix Section 4. Larger VCs with HR > 1 (*p* < 0.05) were present in the posterior oblique beam regions for RADAR, RT01 and Combined, and in the posterior extension of the lateral beams for Combined and RT01. The dose difference maps show patients with incontinence had up to 10 Gy more dose in the lateral beam posterior extension region than patients without incontinence for RT01 and CHHiP, and 9 Gy more for Combined. The LASSO selected HR > 1 voxels in the posterior oblique beam lateral beam posterior lateral beam extension regions for Combined. Only 6 grade ≥ 2 incontinence events were present for CHHiP, thus all voxel HRs were at *p* > 0.05. In summary, patients with increased dose in the vicinity of urethral sphincters and in posterior oblique beam and lateral beam posterior extension regions had a higher incidence of late grade ≥ 2 incontinence.

### Frequency

**Figure 6:**
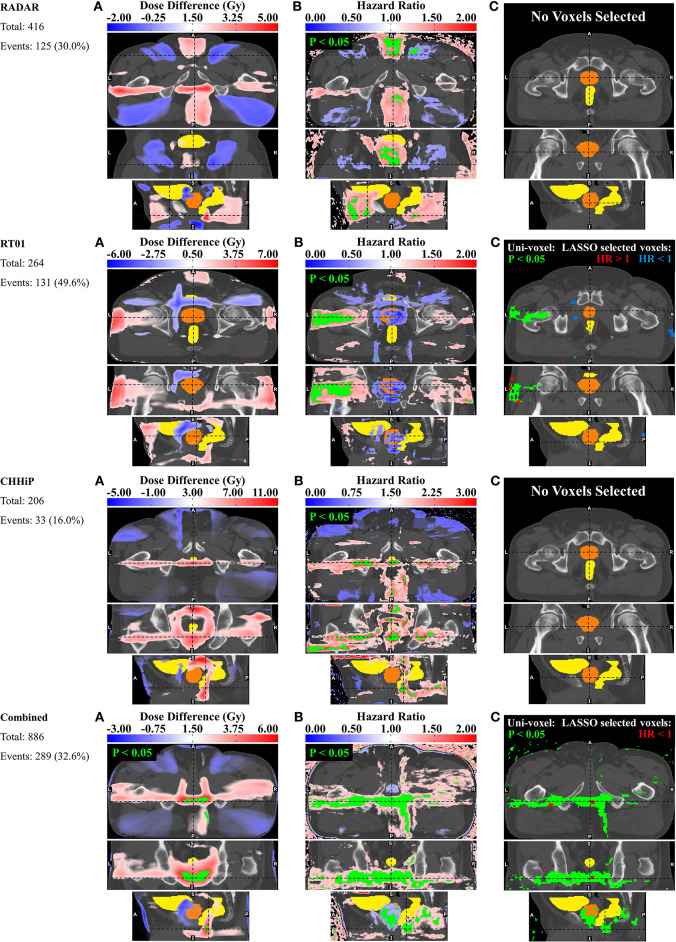
Results for frequency. Corresponding axial, coronal and sagittal slices (top to bottom) of **(A)** mean dose difference maps and regions of significant dose difference determined by permutation test, **(B)** uni-voxel Cox regression HR and *p*-value maps and **(C)** multi-voxel Cox regression LASSO HR maps (with uni-voxel or permutation test *p*-values for comparison), for respective data sets. The slices chosen for display are those which coincide with the most dominant emergent dose-endpoint patterns, indicated in corresponding planes with dashed lines. Tones of red correspond to regions where increased dose is associated with incidence of frequency (HR > 1), while tones of blues correspond to regions where reduced dose is associated with incidence of frequency (HR < 1). The CTV is delineated in orange while the bladder and rectum are delineated in yellow. Anatomical directions left (L), right (R), superior (S), inferior (I), anterior (A), and posterior (P) are also indicated.

Figure 6 shows the results for frequency. The uni-voxel HR maps in this figure reveal the presence of VCs with HR > 1 (*p* < 0.05) in anterior and posterior beam regions inferior to the prostate for RADAR, the left lateral beam for RT01, and (like RADAR) the posterior beam region extending inferiority to the prostate for CHHiP and Combined. Combined showed VCs in the posterior beam region extending inferiorly to the prostate. The permutation test revealed VCs of significant dose difference (*p* < 0.05) in this same region for Combined, where patients with frequency experienced up to 6 Gy more planned dose on average than patients without frequency. RADAR patients with frequency experienced up to 10 Gy more average dose in the same posterior beam region. The LASSO generally selected voxels with HR > 1 in the same regions as the significant HR > 1 VCs found in the uni-voxel maps, for RT01 and Combined, further confirming these associations. In summary, the dominant association revealed was the relationship between patients experiencing more dose in regions extending inferiorly and posteriorly to the prostate and a higher incidence of late grade ≥ 2 frequency.

## Discussion

In this study, quality-assured and reviewed planning data collected in multi-center clinical trials with extensive follow-up was used to derive independent datasets for analysis. Deformable registration of planned dose distributions onto common templates enabled identification of associations between voxel-dose and measures of GU toxicity across the pelvic anatomy. This is the first study to generate dose-GU toxicity relationships of this nature without the assumption that these necessarily occur on or within OARs.

Although no individual voxel-based test in this study addressed every typical shortcoming of voxel- based analyses, each test did address specific problems such that a consistent result across all techniques could be considered independent of these issues. Late genitourinary toxicity differs from late gastrointestinal (GI) toxicity in that the occurrence of late GI toxicity generally reaches a plateau after 3 years post-RT, while late GU toxicity more frequently extends past 3 years (33). This suggests extended follow-up is necessary for the accurate estimation of late GU toxicity. The uni-voxel and multi-voxel Cox regression tests utilized post-treatment time-to-event endpoints with follow-up times extending from approximately 6 to 13 years, enabling an accurate accounting of late GU symptoms. The uni-voxel test controlled for patient baseline characteristics, attempting to remove their confounding influence upon discovered dose-toxicity correlations. The LASSO regression ensured selected voxels, which strongly correlated with GU endpoints, were independent of correlation with other voxels. Incorporating all voxels in the model together accounted for the multiple comparisons problem. The permutation dose difference test similarly accounted for the multiple comparisons problem, while also being the only method of the three that excluded noisy extraneous voxels.

Late grade ≥ 2 dysuria was consistently associated with increased dose to the spongy and membranous urethra regions in this study. Mylona et al. discovered a subregion in the posterior bladder, partially in the trigone, where increased dose was correlated with late grade ≥ 1 dysuria (8). Their study was limited to the bladder and prostatic urethra. Utilizing bladder dose-surface maps, Yahya et al. discovered a similar correlation between late grade ≥ 2 dysuria and increased dose at the posterosuperior bladder surface, near the bladder trigone, however concluded that dysuria was not associated with dose received directly by the trigone (10). The authors predicted, rather, that dose in this region “might also correlate with dose to other organs in the genitourinary system outside the bladder,” alluding to further studies “anticipated to properly investigate this possibility, including the dose to the posterior prostatic urethra and anterior urethra, which includes the membranous and bulbous urethra.” This study has investigated the dose-dysuria relationship in these regions and has indeed found correlation primarily in the membranous and spongy/bulbous urethra and not primarily in the prostatic urethra. The RADAR T3 result did, however, expose some correlation in the prostatic urethra. Thirteen patients in the RADAR cohort included in the dysuria analysis in this study had reported strictures. It is noteworthy that one stricture was found in the prostatic urethra with the rest found in the membranous/spongy urethra. While only 5 of the 13 patients with strictures presented with grade ≥2 dysuria, 8 of the 13 presented with grade ≥ 1 dysuria. Perhaps the membranous/spongy urethra is particularly susceptible to the radiation damage capable of inducing dysuria, such as stricture formation. Dose escalation has been shown to increase urethral strictures in the RADAR cohort (18).

**Figure 7:**
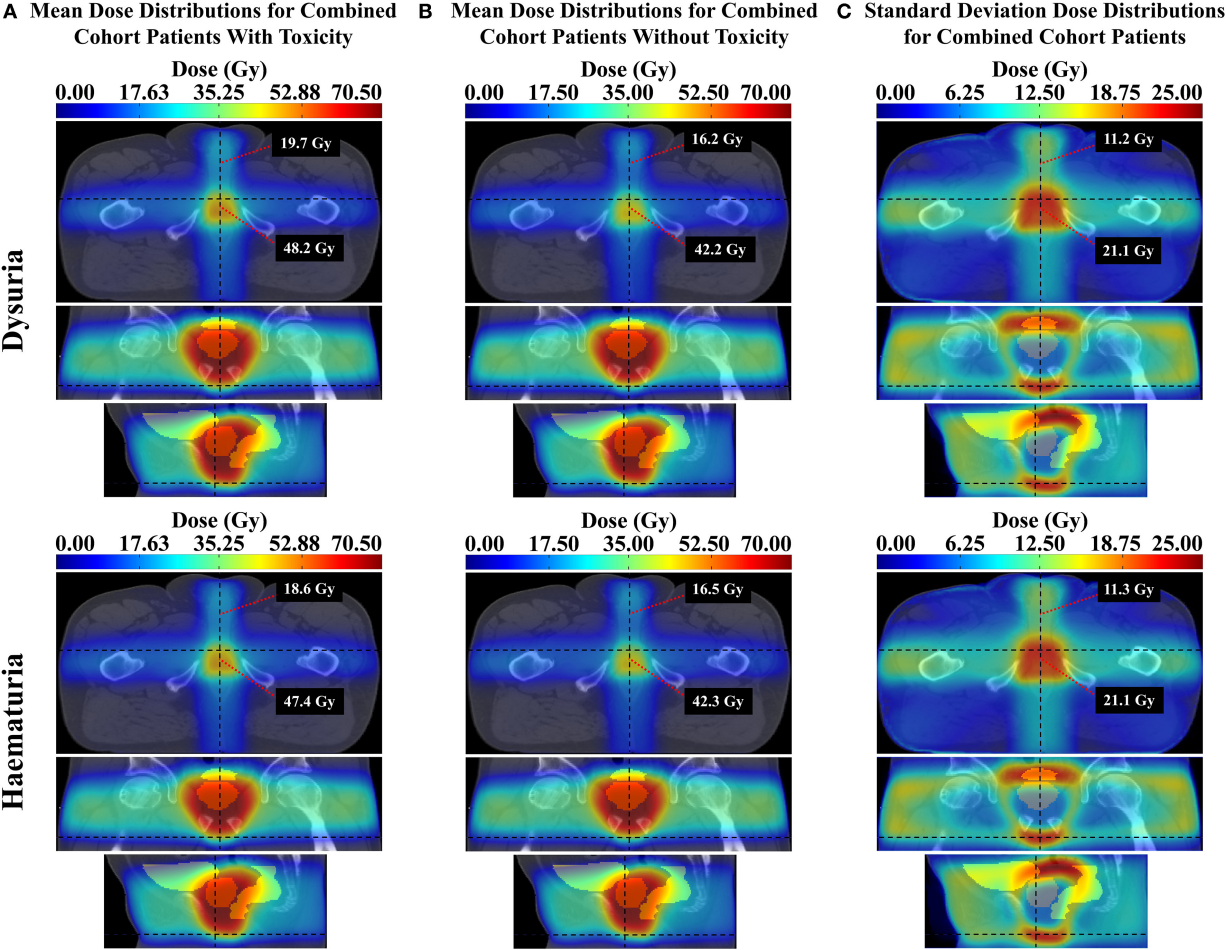
**(A)** Mean dose distributions for patients with the given toxicity in the combined cohort, **(B)** mean dose distributions for patients without the given toxicity in the combined cohort, and **(C)** standard deviation dose distributions maps for patients in the combined cohort dataset for the given toxicity. Each map displays the dose value at a point in the vicinity of the distal spongy urethra (above) and membranous urethra (below). The top row represents the grade ≥2 dysuria dataset and the bottom row the grade ≥1 haematuria dataset.

However, upon examination of the standard deviation dose distributions (see Appendix Section 4 or Figure 7 for just the combined cohort), the maximum dose variation occurs in the membranous/spongy urethra region with minimum variation in the prostatic urethra region, for all trial datasets. This is particularly evident in the T3 standard deviation dose distribution. Therefore, this anterior urethral correlation may have been exposed through sufficient intra-cohort dose-variance in this region, while a potential prostatic urethral correlation may be hidden due to lack of dose variation in the CTV. Figure 7 contains mean dose distributions for the combined cohort displaying dose values in the vicinity of the spongy and membranous urethra, for patients with and without grade ≥ 2 dysuria. Patients with dysuria have a mean dose of 19.7 and 48.2 Gy near the spongy and membranous urethra, respectively. Therefore, this dose-toxicity relationship is occurring in the low dose range at the spongy urethra and intermediate dose range at the membranous urethra. It should be noted that doses <20 Gy are associated with this effect at the spongy urethra. One further hypothesis is that the low dose bath at the distal spongy urethra may reduce the capacity of stem cells to migrate back to the more heavily irradiated prostatic (or even membranous) urethra where they would facilitate urethral healing (34, 35). In conclusion, there is strong evidence that radiation dose to the urethra is associated with resulting dysuria. The membranous and spongy urethra may be particularly susceptible, however dose to the prostatic urethra is likely to be related to dysuria as well. Limiting dose to the spongy urethra may be more realistic as the prostatic urethra resides in the high dose region. Future studies delineating the prostatic/membranous/spongy urethra and investigating the dose-volume-dysuria relationship in these regions may further characterize urethral dose sensitivity.

Late grade ≥ 1 haematuria was similarly associated with increased dose to the spongy and membranous urethra regions. Urinary bleeding has typically related to the high dose region of the bladder (14, 36–38) where the bladder neck and trigone reside, and has been observed in the bladder neck and trigone at cystoscopy (8). Yahya et al. (10), however, in agreement with the superior bladder subregion found by Mylona et al. (8), determined that late haematuria was associated with dose to the anterosuperior regions of the bladder, and concluded that haematuria was not a result of dose to the trigone or bladder neck, but rather to tissue damage in the bladder wall. Although Inokuchi et al. investigated and found no dose-volume association with haematuria at the prostatic urethra (14), no study to date has investigated dose-haematuria association at the membranous or spongy urethra. To the best of the author's knowledge this is the first study to have included these regions of the urethra in localized dose-toxicity analysis. As a result, an association with haematuria has been found in the extra-prostatic urethra, contrary to the general pattern. This is not beyond the scope of current evidence, however, as haematuria can be caused by urinary tract infection and strictures (39). As previously discussed, urethral strictures were present in the cohort and can result from urethral radiation damage. Considering patients in the subset of the RADAR cohort included in the haematuria analysis, 9 out of the 12 patients with reported spongy/membranous urethral strictures also had grade ≥ 1 haematuria. It is also plausible that radiation damage to the anterior urethra can cause inflammation leading to urinary tract infection. As demonstrated for dysuria, Figure 7 shows that this haematuria effect is associated with doses lower than 20 Gy at the spongy urethra. Therefore, reduced stem cell migration in response to the low dose bath at the spongy urethra may also be contributing to this effect. Due to these considerations and the similar association found with dysuria, limiting dose to the anterior urethra may substantially reduce the incidence of these two prominent urinary toxicities. Spongy urethral dose could be reduced by taping down the penis to the thigh. Or perhaps by using two anterior oblique beams instead of a single anterior beam.

It should also be acknowledged that the above dysuria and haematuria dose relationships were found in 3D-CRT patients (from the RADAR and RT01 trials). In the current era, almost all patients are treated with IMRT or VMAT which result in more conformal dose distributions in comparison to 3D-CRT. It is therefore very likely that the majority of contemporary patients would receive less spongy urethral dose, perhaps eliminating the need for the application of dose constraints or any dose reduction scheme. This is even evident in the current study, with CHHiP patients (treated with IMRT) experiencing less urethral dose correlation and less events corresponding to both dysuria and haematuria. It is also noteworthy that applying dose constraints to the membranous urethra, which begins proximally at the prostatic apex, may not be feasible due to its proximity to the high dose PTV region. The reader is reminded that the primary purpose of this study was not to discover dose constraints but to explore the underlying causal relationships between localized dose and toxicity without the assumption that these relationships necessarily occurred at OAR sites (from which future analyses may derive dose constraints if appropriate—see the third final paragraph of this discussion).

Increased dose in the external and internal urethral sphincter and in the posterior oblique beams was shown to correlate with late grade ≥ 2 incontinence. It is established that urinary incontinence can (40), and most commonly does (41), result from urethral sphincter malfunctioning. Although both the internal and external sphincters are involved in maintaining continence, the internal sphincter is of primary importance (42). Mylona et al. found a predictive subregion in the prostatic urethra for incontinence, concluding this was related to damage to the urethral sphincter (8). Yahya et al. found an association between incontinence and dose to the posteroinferior bladder at the trigone, suggesting this was likely related to dose received by the internal sphincter (10). The sphincter muscles may be scarred by irradiation, or dose to the nearby bladder neck may increase ischemia and fibrosis and thus incontinence due to internal sphincter damage (10). The association with the posterior oblique beams may be a surrogate for dose directly to the sphincters, although no direct evidence for this has been discovered. It is recommended that clinicians be aware of the potential radiation damage to the sphincters, while recognizing they do coincide with the high dose region, and that a large scale clinical trial did not reveal increasing incidence of incontinence with dose escalation (43).

Late grade ≥ 2 frequency was associated with dose extending inferiorly and posteriorly to the prostate and rectum. This may indicate that patients treated with a posterior beam extending inferiorly beyond the rectum had a higher incidence of frequency. This result is largely unintuitive and difficult to rationalize. It is noteworthy that Mylona et al. could not demonstrate a dosimetric association with urinary frequency (8).

The relationships presented here are correlations that may or may not represent anatomically-localized physiological dose-toxicity associations. The low number of toxicity events, namely <10% of the cohort for 7 of the 16 datasets, should reinforce this suspicion. Only the uni-voxel Cox regression accounted for intrinsic patient factors, and these represent only a sample of possible patient cofactors that could confound the associations. To ensure dose-toxicity relationships are independent of a given patient factor, separating the cohort into this factor's subgroups prior to analysis is necessary. This, however, would reduce power, requiring a larger cohort to establish statically meaningful associations. Furthermore, these relationships must be interpreted in light of the differences in dosemaps from the three trials. For example, the mean dose distributions from respective trials (see Appendix Section 4) indicate that the average CHHiP distribution is more conformal than that of RADAR or RT01. This is consistent with the fact that CHHiP patients received IMRT instead of 3D-CRT for RADAR and RT01, and may explain why the number of toxicity events were lower and correlation patterns were much weaker in CHHiP datasets. It must also be noted that the follow-up times were not identical between the datasets derived from the three trials. For example, datasets from the RADAR trial included follow-up data at 9 and 15 months post-treatment while the datasets from the RT01 and CHHiP trails did. Defining the endpoints differently in this way may bias the comparison and therefore it is that data from the same timepoints are used in generating endpoints when comparing applying analysis to different trials with the goal of comparing results. Finally, it is recognized that the results from the combined cohort (for all toxicity endpoints) are biased toward the RADAR dataset as RADAR patients comprise a higher proportion of this dataset than patients from the other two trials. The goal of the combined analysis was to maximize the statistical power available for each toxicity endpoint and observe the resulting dose-toxicity patterns. The aforementioned bias was therefore accepted as necessary to achieve this goal. Although, in future analyses, if an adequate number of patients are available, it is recommended that, in addition to combining all patients together, an equal number of patients from each trial be included in the combined dataset (or a form of normalization employed) to remove bias from any individual trial dataset. Comparing results from a combined cohort with those from a balanced (or normalized cohort) would be useful in discerning the bias introduced from the dominant dataset.

The permutation test is quite conservative. In the dose difference comparison between patients with and without an event pertaining to a given endpoint, it applies a global threshold that cannot identify local maxima of dose difference. Also, due to the large number of voxels compared, in order to adequately account for the multiple statistical testing problem this threshold can be quite high, and therefore may exclude not only local regions of significant dose difference but also global regions. Hence only large and statistically strong global dose differences can be identified (and therefore *p*-value thresholds up to *p* < 0.3 were used). This could explain why, across all datasets and endpoints, only in one dataset (Combined for frequency) was a region of statistically significant dose difference discovered by this test. A test more sensitive in identifying local maxima, such as a threshold-free cluster enhancement test (44), may be appropriate for further voxel-based analyses. Palorini et al. (12) outline further reasons for being wary of a straightforward application of the permutation test in the context of bladder dose surface maps. These include (1) the distribution of *t*-scores obtained in each pixel being significantly different from the null distribution (invalidating the test's assumption of a universal null hypothesis), and (2) macro regions of heterogenous voxel dose skewness reducing the probability of regions with less skewness of registering a significant dose difference. Point (1) implies that the test be overly restrictive, while (2) indicates the need for possibly dividing the dose distribution into regions of relatively uniform skewness and repeating the test on each region separately. This may be an appropriate future directive for using this test in the context of voxel-based analysis.

The assumption that planned dose is equivalent to delivered dose, which differ in reality (45), is a major limitation of this study. It has been shown that delivered dose can be a better predictor of rectal toxicity than planned dose (46). As the agreement between planned and delivered dose improves, or delivered dose becomes more easily measurable, voxel-based dose analyses will be more effective in identifying anatomically localized dose-toxicity relationships. Cone beam CT daily imaging, for example, could be used to measure cumulative delivered dose across the course of treatment (47). Furthermore, all voxel-based tests in this analysis were applied throughout the entire pelvic region, including a broad range of late-responding normal tissues. An alpha/beta value of 3 was chosen as it has been regarded as generally representative of all late responding normal tissues (48). It is acknowledged, however, that different normal tissues respond differently with respect to different toxicities, thus resulting in different alpha/beta values. Therefore, an appropriate future direction would be to test the sensitivity of results to different alpha/beta values, particularly with respect to the different urinary toxicities. Another limitation could be the registration accuracy and the suitability of the choice of exemplar and anti-exemplar. The anatomical localization of the emergent dose-toxicity patterns is directly dependent on registration accuracy. A perfect registration would ensure the identified patterns are in fact occurring at the presumed anatomical sites. Diversity in the distribution of dose across each cohort is also limiting, as the mean dose distributions are approximately 3 or 4 field treatments in all datasets (see Appendix Section 4 for mean and standard deviation dose distributions). Greater diversity in technique will enable more generalizable feature selection. Differences in diversity between trials may also account for lack of consistency in results across trials. For example, RADAR treatments were allowed any combination of 3 or more beams, while RT01 treatments were restricted to 3 or 4 beams in the anterior/lateral/posterior directions. This has led to differences in the spatial distribution of dose variation between trial cohorts, resulting in different potential sites with sufficient variation for exposing dose-toxicity associations.

Incorporating the voxel-based evidence into normal tissue complication probability (NTCP) models may facilitate translation of these results into clinical practice. Palma et al. have derived a new NTCP philosophy to include voxel-based evidence of OAR radio-sensitivity (49). Incorporating the evidence of OAR sensitivity from this study into a model like this could result in reduced toxicity for patients when applied to treatment planning. It is also acknowledged that the majority of evidence discovered in this study was from 3D-CRT patients. Therefore, the methods here may need to be applied to a larger cohort of patients treated with contemporary techniques before translation is made to the clinic.

This study focused on urinary specific and not toxicities related to sexual function. An exploration of the relationship between erectile dysfunction (ED) and dose in a voxel-based manner is recommend for future analyses. Additionally, the relationship between ED and dose to the penile bulb was studied elsewhere for the RT01 and CHHiP patients (50, 51). Finally, the study of the relationship between ED and dose is problematic in cohorts that use additional ADT due to the impact of ADT on various measures of sexual function including ED (52–54). The RADAR trial, from which our primary dataset was derived, is one such cohort. Additionally, it has previously been shown that ADT did not increase urinary dysfunction (including dysuria, haematuria, frequency and incontinence) in the RADAR cohort (55). Similar studies have not been performed for the RT01 and CHHiP cohorts, however, as trial arms did not vary in terms of ADT duration. The impact of ADT may therefore be a confound in the datasets derived from RT01 and CHHiP.

This was the first study performing a full voxel-based analysis of dose-urinary toxicity relationships in the entire pelvic anatomy, without the assumption that these occurred exclusively at OAR sites. Associations between late dysuria and haematuria and dose to the spongy and membranous urethra have been newly identified, while dose to the urinary sphincters and resulting incontinence has confirmed the idea that radiation damage at the sphincter can cause incontinence.

## Data Availability Statement

The datasets for this article are not publicly available because of ethical restrictions placed on patient data derived from clinical trials. Requests to access the datasets should be directed to Marco Marcello, 20739859@student.uwa.edu.au.

## Ethics Statement

The studies involving human participants were reviewed and approved by the Hunter New England Human Research Ethics Committee Trial ID 03/06/11/3.02 for the RADAR trial, the North Thames Multi-center Research Ethics Committee number MREC/97/2/16 for the RT01 trial, and the London Multi-center Research Ethics Committee number 04/MRE02/10 for the CHHiP trial. The patients/participants provided their written informed consent to participate in this study.

## Author Contributions

All contributors met the criteria required to be considered a qualified author. All authors approved of the final submitted version of the manuscript and agree to be accountable for all aspects of the work. MM performed all statistical analyses, processed the results, and was the primary author of the manuscript and supplementary material. JD, AH, and ME provided expert information on the RADAR trial and contributed to the editing of the manuscript. They also assisted in acquisition of the RADAR trial data. MS did all of the above, however for the RT01 trial data. DD and SG did the same for both the RT01 and CHHiP trial data. EH for just the CHHiP trial data. AK performed the dose distribution registrations and provided technical support in displaying the results on CT templates. AS provided expert information assisting with aspects of the statistical analysis. PG, LH, MJ, and DR provided expert information in regards to the methodology and interpretation of results, and contributed to the editing of the manuscript. JD provided expert information in regards to the registration process and interpretation of results, and contributed to the editing of the manuscript. DJ assisted in acquisition of the RADAR trial data.

## Conflict of Interest

DD discloses that his employer, the Institute of Cancer Research, receives royalty income from Abiraterone and receives a share of this income through the ICR's Rewards to Discoverers Scheme. MS reports grants from Health Data Research UK, during the conduct of the study; personal fees from Lilly Oncology and Janssen, grants and non-financial support from Astellas, Clovis Oncology, Janssen, Novartis, Pfizer, and Sanofi-Aventis, outside the submitted work. EH reports grants from Cancer Research UK, during the conduct of the study; grants from Accuray Inc., outside the submitted work. ME reports grants from Australian National Health and Medical Research Council, during the conduct of the study. The remaining authors declare that the research was conducted in the absence of any commercial or financial relationships that could be construed as a potential conflict of interest.
