# Supplementary material for: Increased Dose to Organs in Urinary Tract Associates With Measures of Genitourinary Toxicity in Pooled Voxel-Based Analysis of 3 Randomized Phase III Trials
Source: Front Oncol. 2020 Jul 22;10:1174. doi: 10.3389/fonc.2020.01174 (PMC7387667; doi:10.3389/fonc.2020.01174)
Supplement: Supplementary file 1 [file Data_Sheet_1.PDF]

## Appendix

### *Section 1: Automated Control Variable Selection*

For each data set (RADAR, RT01, CHHiP and Combined), four genitourinary toxicity endpoints were tested for association with dose. Prior to testing this association using the three voxel-based analyses outlined in the body of the paper, significant baseline variables were established for use in the uni-voxel Cox regression. These were chosen using an automated variable selection algorithm on SAS version 9.4. Specifically, it is the 'phreg' function utilising the 'stepwise' selection option.

This algorithm combined all baseline variables (age, prescribed dose, disease risk, cancer stage, baseline PSA concentration and number of treatment beams) into a Cox proportional hazards model testing for the association between these variables and a given endpoint (dysuria, haematuria etc). The algorithm then performs a stepwise removal of variables that are not showing a statistically significant association with the endpoint at the $p < 0.05$ level, until only significant variables remain. These variables were chosen as controls in each corresponding uni-voxel Cox regression model, to remove their confounding influence on the relationship between the given endpoint and voxel dose.

In order to ensure each model is adequately powered, it is permissible to include a maximum of 1 variable per 10 endpoint events (1). Therefore, if more than 1 control variable was selected per 10 events then the most significant control variables were chosen up to the permissible limit. Results for all endpoints in all datasets are shown in Table A1.

***Supplementary Table 1*** *Results showing the significant control variables selected by the automated control variable selection algorithm on SAS. For each selected control, the corresponding hazard ratio with 95% confidence interval and p-value is included. The number of events is included in brackets next to each endpoint label. Results are shown for every endpoint in all datasets.*

| | Significant control variables | | | | | |
|---|---|---|---|---|---|---|
| Data set and endpoint, with number of events | Age | Dose | Risk | Stage | PSA | Beams |
| RADAR_DYS (79) | | 74 Gy vs 66 Gy:<br>2.07 (0.998,4.287), p = 0.051<br>Overall: p = 0.016 | | | | |
| RADAR_HAEM (86) | | | | 2.83 (1.06,7.55), p = 0.038 | | |
| RADAR_INC (24) | | | | | | 5 beams vs 3 beams:<br>3.59 (0.97,13.31), p = 0.057<br>Overall: p < 0.001 |
| RADAR_FREQ (125) | | | | None | | |
| | Age | Dose | Risk | Stage | PSA | Beams |
| RT01_DYS (36) | | | 2.64 (1.03,6.78), p = 0.044 | | | |
| RT01_HAEM (52) | | | | None | | |
| RT01_INC (26) | | | | None | | |
| RT01_FREQ (131) | | 1.45 (1.03,2.04), p = 0.035 | | | | |
| | Age | Dose | Risk | Stage | PSA | Beams |
| CHHiP_DYS (11) | 3.87 (1.17,12.80), p = 0.026 | | | 0.19 (0.04,0.89), p = 0.035 | | |
| CHHiP_HAEM (21) | None | | | | | |
| CHHiP_INC (6) | | | | 5.94 (1.09,32.55), p = 0.040 | | |
| CHHiP_FREQ (33) | None | | | | | |
| | Age | Dose | Risk | Stage | PSA | Beams |
| COMBINED_DYS (126) | 0.67 (0.47,0.96), p = 0.029 | 1.73 (1.17,2.55), p = 0.006 | | | | |
| COMBINED_HAEM (159) | | | 1.84 (1.18,2.89), p = 0.008 | | | |
| COMBINED_INC (56) | | | | | | 1.95 (1.16,3.30), p = 0.012 |
| COMBINED_FREQ (289) | | 1.56 (1.22,2.00), p < 0.001 | | | | |

***Section 2: Registration Details***

***Registration Pipelines***

Pipeline 1 (for T1 and T2):

1. Perform the pre-processing applied to images prior to registration for clustering.
   a. CT to HU parameter estimation.
   b. Rotations to ensure all images are oriented the same way.
   c. Slice gap correction to correct for uneven slice gaps.
   d. Body masking to remove couch and external artefacts.
   e. Cropping to the superior most of the superior extent of the bladder and to the inferior extent of the anorectum + a 1cm margin.
2. Rigid registration using an inverse consistent block matching rigid registration algorithm called Mirror (2) with the normalised correlation as the optimisation metric was performed on pre-processed CT images.
3. Deformable registration using an Insight Segmentation and Registration Toolkit (3) implementation of the Diffeomorphic Demons registration algorithm (4) using 3 pyramid levels.

Pipeline 2 (for T3) (extra steps were taken to improve registration accuracy in the CTV region):

1. Perform the pre-processing applied to images prior to registration for clustering.
   a. CT to HU parameter estimation.
   b. Rotations to ensure all images are oriented the same way.
   c. Slice gap correction to correct for uneven slice gaps.
   d. Body masking to remove couch and external artefacts.
   e. Cropping to the superior most of the superior extent of the bladder or the location where the hips and spine first join and to the inferior extent of the anorectum + a 1cm margin.

f. Fill air regions within the body using values from surrounding tissues + Gaussian noise.
g. Remove seeds for all images that had them (19 RADAR images and T3).
h. Interpolate to a resolution of 1mm between axial slices using tri-linear interpolation.
i. Perform 1 pixel median filtering to decrease image artefacts.
j. Perform Gaussian mixture modelling (GMM) to separate 4 classes of tissues; background (GMM0), fat (GMM1), water & muscle (GMM2), and bone (GMM3).
k. Threshold GMM3 to create a binary bone mask image.

2. Rigid registration using an inverse consistent block matching rigid registration algorithm called Mirror (2) with the normalised correlation as the optimisation metric was performed on bone mask images with the registration then applied to GMM2 images and CTV structures where they were available.
3. Crop rigidly registered GMM2 images to the region of the fixed image (T3) CTV + a 5cm margin in 3 dimensions.
4. Deformable registration using an Insight Segmentation and Registration Toolkit (3) implementation of the Diffeomorphic Demons registration algorithm (4):
    a. If a CTV was available:
        i. On the CTVs using 4 pyramid levels.
        ii. On the cropped GMM2 images with the deformation field using 2 pyramid levels and with the deformation field initialised using the results of the deformable CTV registration from step i.
    b. If no CTV was available: on the cropped GMM2 images using 3 pyramid levels.

Note that pipeline 1 and 2 use the first and second cropping methods respectively, according to the registration processes outlined by Kennedy et al (5).

*Registration Templates*

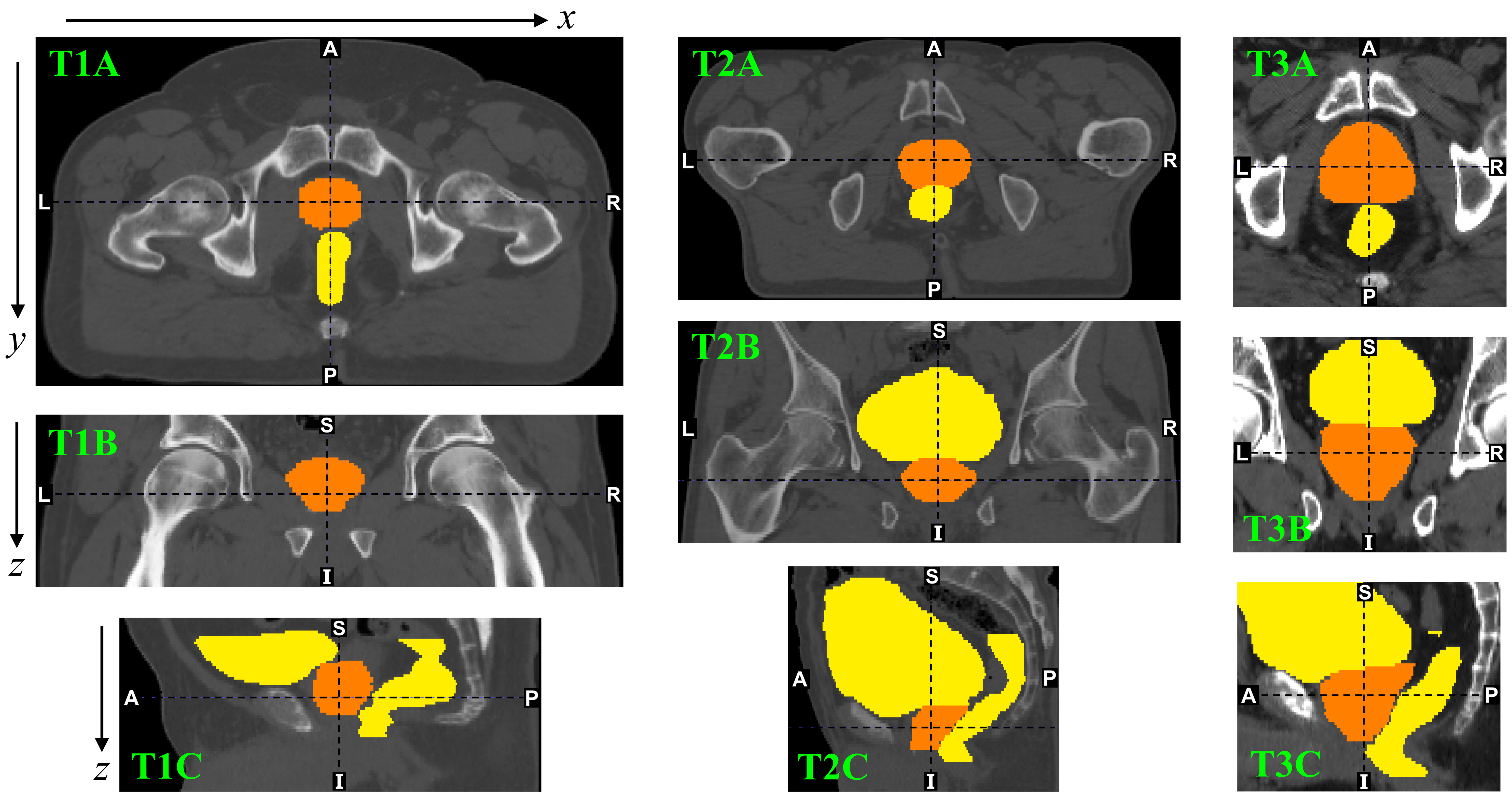


***Supplementary Figure 1*** *Registration templates T1, T2 and T3, onto which 3D dose distributions were mapped. A, B and C refer to axial, coronal and sagittal planes respectively. A, P, L, R, S, I refer to anatomic directions anterior, posterior, left, right, superior, inferior respectively. Bladder and rectum delineations are in yellow while the CTV delineation is in orange. Bladder and rectum are both displaced in yellow to avoid clashes of colour once statistical maps are imposed.*

The number of voxels in (and dimensions of) each CT registration template and corresponding dose distributions are as follows:

T1: (x, y, z) = 332 x 249 x 64 voxels = 388.108 x 291.081 x 128.000 mm, voxel size: (x, y, z) = 1.169 x 1.169 x 2.000 = 2.733 cubic mm
T2: (x, y, z) = 327 x 178 x 76 voxels = 383.244 x 208.616 x 190.000 mm, voxel size: (x, y, z) = 1.172 x 1.172 x 2.500 = 3.434 cubic mm
T3: (x, y, z) = 132 x 130 x 129 voxels = 164.208 x 161.720 x 129.000 mm, voxel size: (x, y, z) = 1.244 x 1.244 x 1.000 = 1.548 cubic mm

Due to the large number of total voxels in T1 and T2, all corresponding analyses used dose distributions which sampled 1 in 2 voxels. For T3, every voxel was used.

*Section 3: Full Results for All Voxel-Based Analyses*

***Supplementary Figure 2*** *Example results with labels and corresponding information required to interpret the results figures on the following pages.*

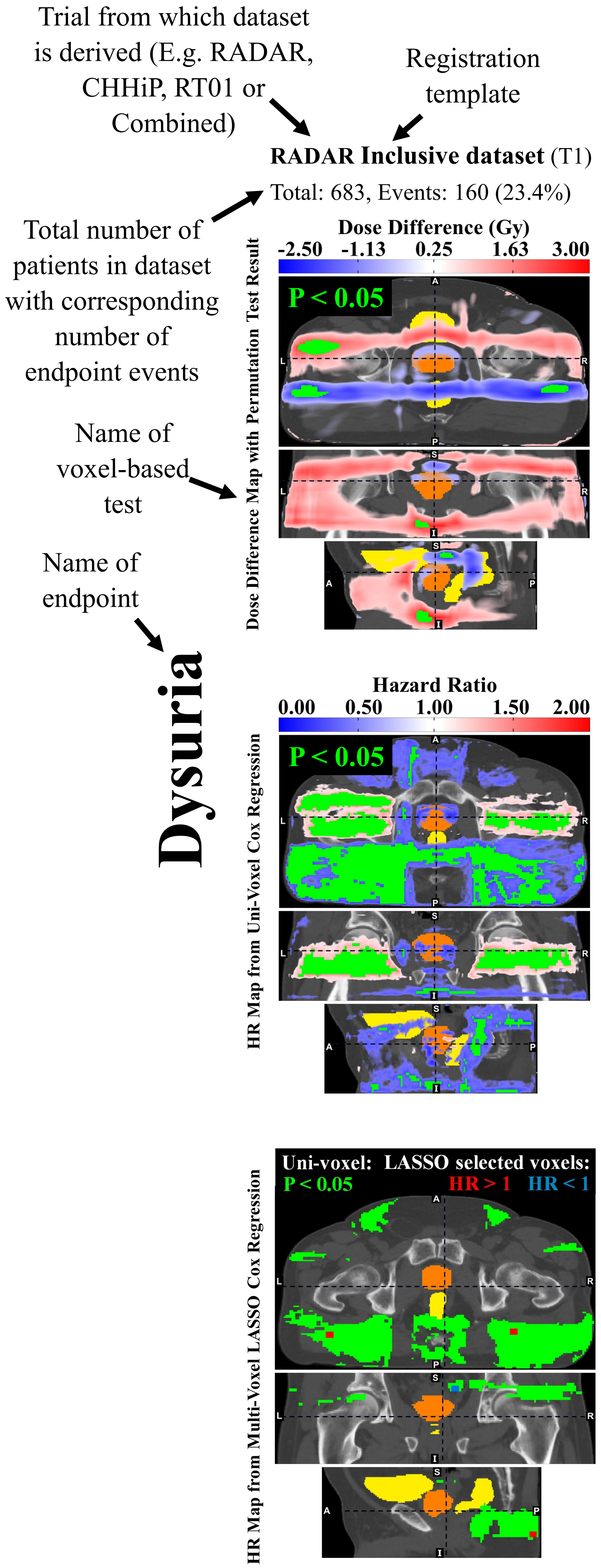


**Dose difference map and permutation test region**

The dose difference map shows the difference in planned voxel dose between the mean dose distributions of patients who experienced an endpoint event and those who didn't (e.g. in the image we see regions along the posterior lateral beam boundary near the patient skin where patients with a dysuria event had up to 2-2.5 Gy *less* planned dose than patients without a dysuria event). The permutation test results, in the form of a thresholded p-value map, are then imposed on top of this. These show regions where the dose difference is significant (at the $p < 0.05$ level) according to the permutation test. However, almost all permutation tests found no regions of significant difference, so the dose difference maps are displayed on their own. Note that all maps show corresponding axial, sagittal and coronal planes, from top to bottom.

**Hazard ratio (HR) map and corresponding p-value map from the uni-voxel Cox regression test**

The p-value map is imposed on the HR map. Warmer colours (toward the red end of the colour spectrum) generally represent regions where the voxel-based HR is greater than 1, which implies *higher* dose is correlated with a higher incidence of the endpoint in these regions. Colder colours (toward the blue end of the colour spectrum) correspond to regions where HR < 1 and therefore *lower* dose is correlated with a higher incidence of the endpoint. The p-value map, in fluoro green, shows regions where the HRs were significantly greater than or less than 1 at the $p < 0.05$ level.

**HR maps of voxels selected by multi-voxel LASSO Cox regression test**

The HR > 1 red voxels are those selected by the model in indication of a strong correlation between *higher* dose and a higher incidence of the endpoint, independent of correlation with other voxels. Similarly, the HR < 1 blue voxels are those selected by the model in indication of a strong correlation between *lower* dose and a higher incidence of the endpoint. The corresponding uni-voxel Cox thresholded ($p < 0.05$) p-value map is co-displayed for direct comparison. In many cases, only voxels with HR > 1 or HR < 1 were selected by the model. Sometimes no voxels were selected or those selected were considered noise as they were outside the patient, and this is indicated with a 'No Voxels Selected' sign.

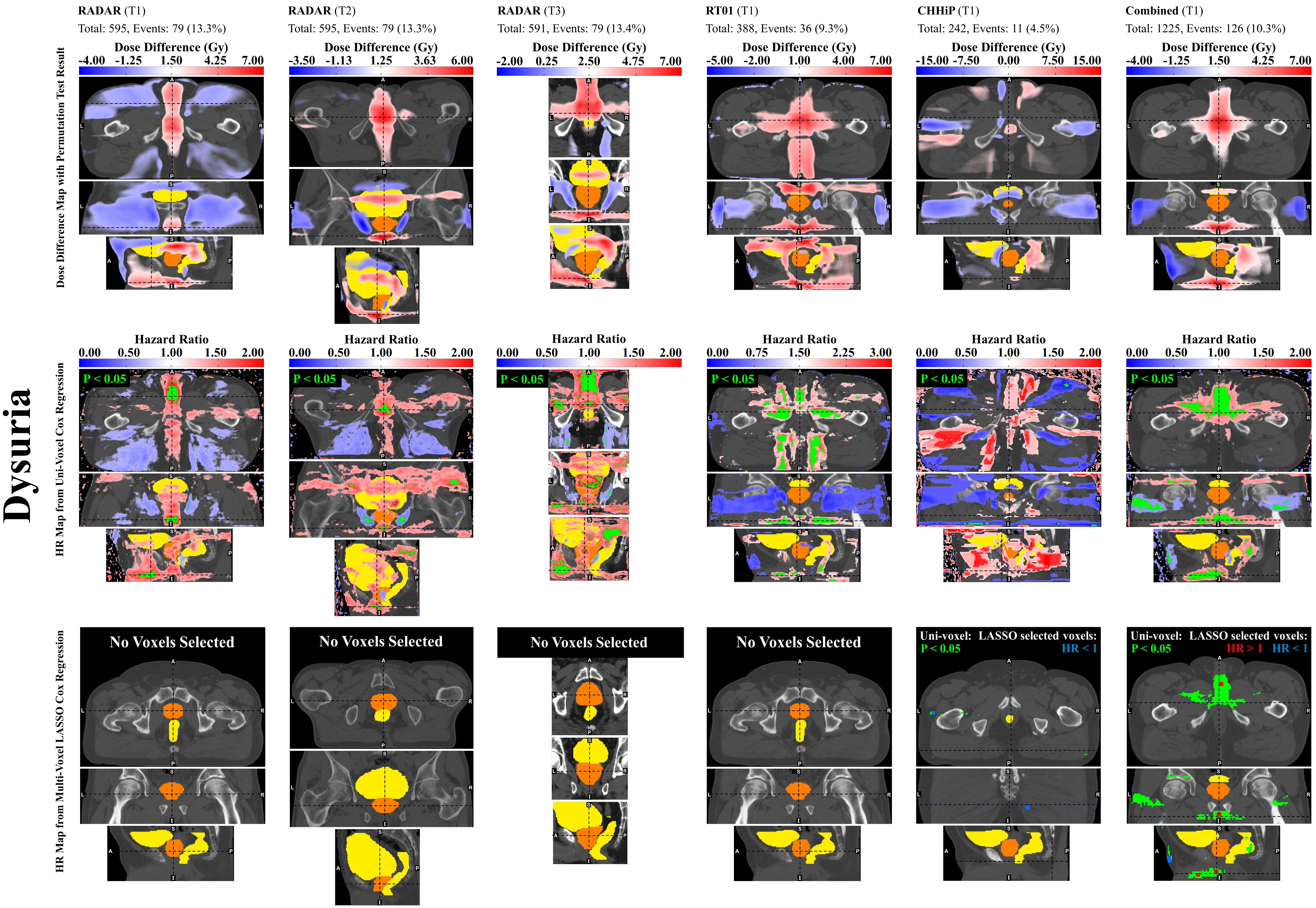

Dysuria
Dose Difference Map with Permutation Test Result
HR Map from Uni-Voxel Cox Regression
HR Map from Multi-Voxel LASSO Cox Regression
RADAR (T1)
Total: 595, Events: 79 (13.3%)
RADAR (T2)
Total: 595, Events: 79 (13.3%)
RADAR (T3)
Total: 591, Events: 79 (13.4%)
RT01 (T1)
Total: 388, Events: 36 (9.3%)
CHHiP (T1)
Total: 242, Events: 11 (4.5%)
Combined (T1)
Total: 1225, Events: 126 (10.3%)
Dose Difference (Gy)
-4.00 -1.25 1.50 4.25 7.00
-3.50 -1.13 1.25 3.63 6.00
-2.00 0.25 2.50 4.75 7.00
-5.00 -2.00 1.00 4.00 7.00
-15.00 -7.50 0.00 7.50 15.00
-4.00 -1.25 1.50 4.25 7.00
Hazard Ratio
0.00 0.50 1.00 1.50 2.00
0.00 0.75 1.50 2.25 3.00
P < 0.05
No Voxels Selected
Uni-voxel: LASSO selected voxels:
P < 0.05
HR > 1
HR < 1

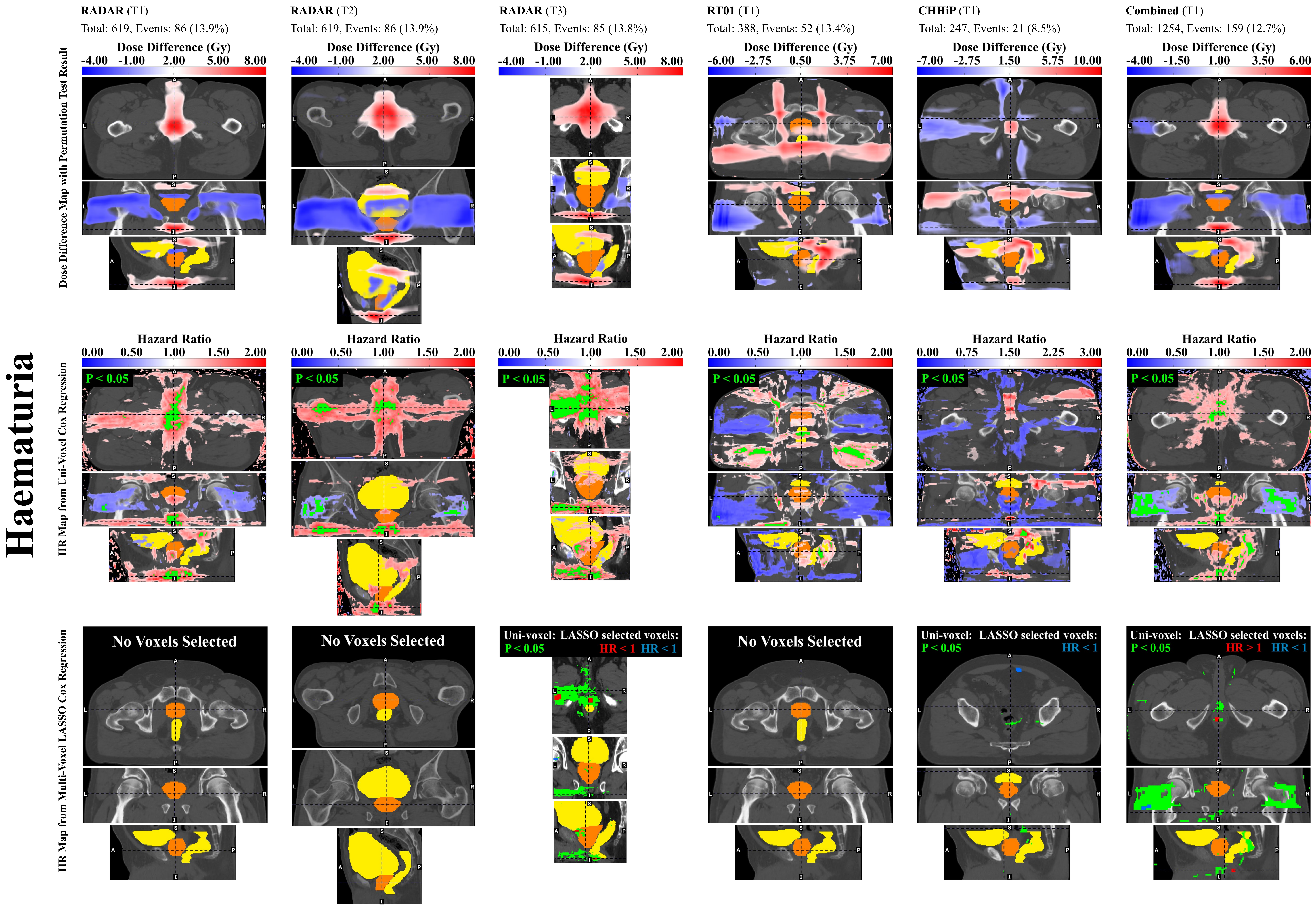

Haematuria
Dose Difference Map with Permutation Test Result
HR Map from Uni-Voxel Cox Regression
HR Map from Multi-Voxel LASSO Cox Regression
RADAR (T1)
Total: 619, Events: 86 (13.9%)
Dose Difference (Gy)
-4.00 -1.00 2.00 5.00 8.00
RADAR (T2)
Total: 619, Events: 86 (13.9%)
Dose Difference (Gy)
-4.00 -1.00 2.00 5.00 8.00
RADAR (T3)
Total: 615, Events: 85 (13.8%)
Dose Difference (Gy)
-4.00 -1.00 2.00 5.00 8.00
RT01 (T1)
Total: 388, Events: 52 (13.4%)
Dose Difference (Gy)
-6.00 -2.75 0.50 3.75 7.00
CHHiP (T1)
Total: 247, Events: 21 (8.5%)
Dose Difference (Gy)
-7.00 -2.75 1.50 5.75 10.00
Combined (T1)
Total: 1254, Events: 159 (12.7%)
Dose Difference (Gy)
-4.00 -1.50 1.00 3.50 6.00
Hazard Ratio
0.00 0.50 1.00 1.50 2.00
P < 0.05
Hazard Ratio
0.00 0.50 1.00 1.50 2.00
P < 0.05
Hazard Ratio
0.00 0.50 1.00 1.50 2.00
P < 0.05
Hazard Ratio
0.00 0.50 1.00 1.50 2.00
P < 0.05
Hazard Ratio
0.00 0.75 1.50 2.25 3.00
P < 0.05
Hazard Ratio
0.00 0.50 1.00 1.50 2.00
P < 0.05
No Voxels Selected
No Voxels Selected
Uni-voxel: LASSO selected voxels:
P < 0.05 HR < 1 HR < 1
No Voxels Selected
Uni-voxel: LASSO selected voxels:
P < 0.05 HR < 1
Uni-voxel: LASSO selected voxels:
P < 0.05 HR > 1 HR < 1

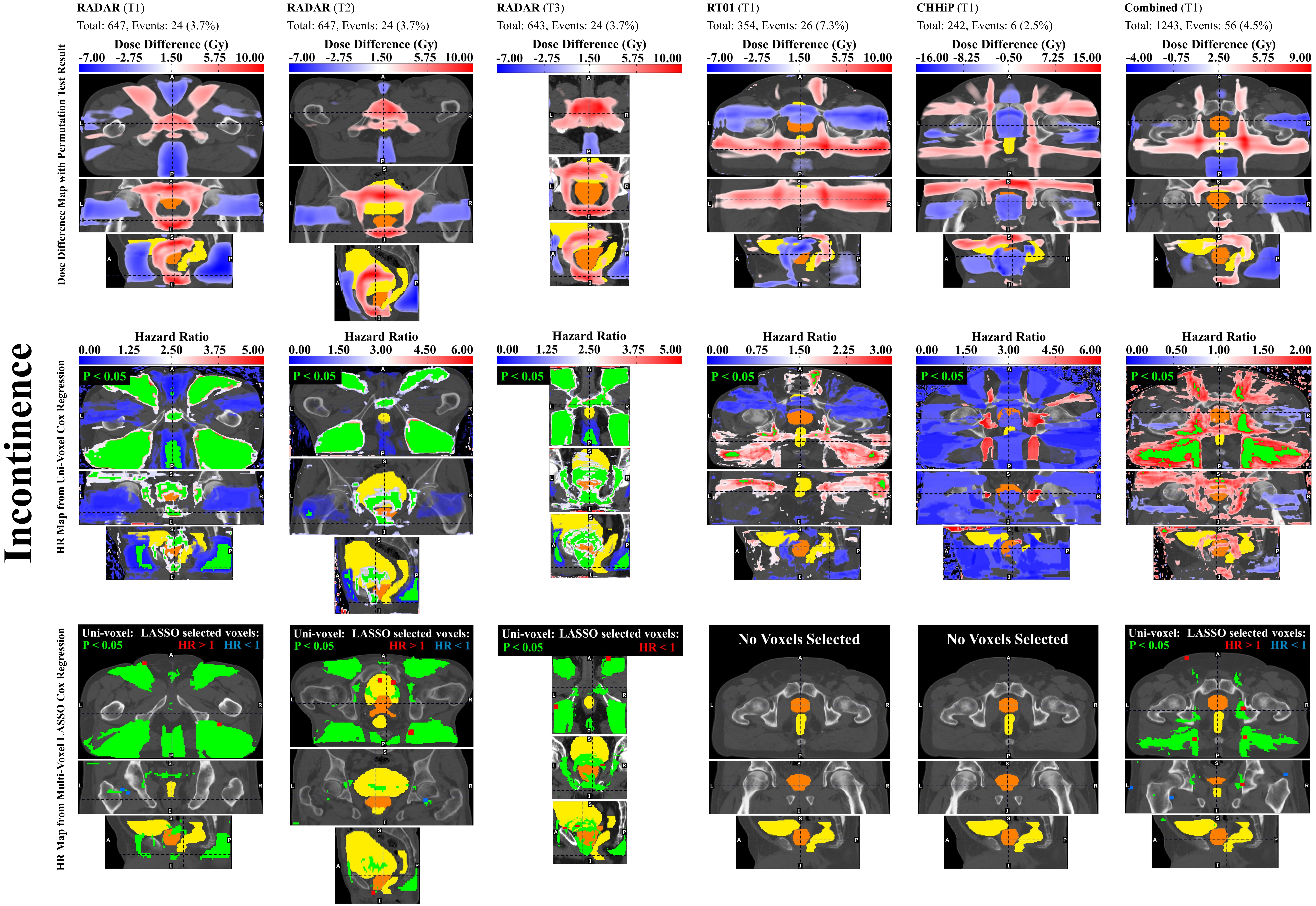

Incontinence
RADAR (T1)
Total: 647, Events: 24 (3.7%)
RADAR (T2)
Total: 647, Events: 24 (3.7%)
RADAR (T3)
Total: 643, Events: 24 (3.7%)
RT01 (T1)
Total: 354, Events: 26 (7.3%)
CHHiP (T1)
Total: 242, Events: 6 (2.5%)
Combined (T1)
Total: 1243, Events: 56 (4.5%)
Dose Difference Map with Permutation Test Result
Dose Difference (Gy)
-7.00 -2.75 1.50 5.75 10.00
-16.00 -8.25 -0.50 7.25 15.00
-4.00 -0.75 2.50 5.75 9.00
HR Map from Uni-Voxel Cox Regression
Hazard Ratio
0.00 1.25 2.50 3.75 5.00
0.00 1.50 3.00 4.50 6.00
0.00 0.75 1.50 2.25 3.00
0.00 0.50 1.00 1.50 2.00
P < 0.05
HR Map from Multi-Voxel LASSO Cox Regression
Uni-voxel: LASSO selected voxels:
P < 0.05
HR > 1
HR < 1
No Voxels Selected
A
P
L
R
S
I

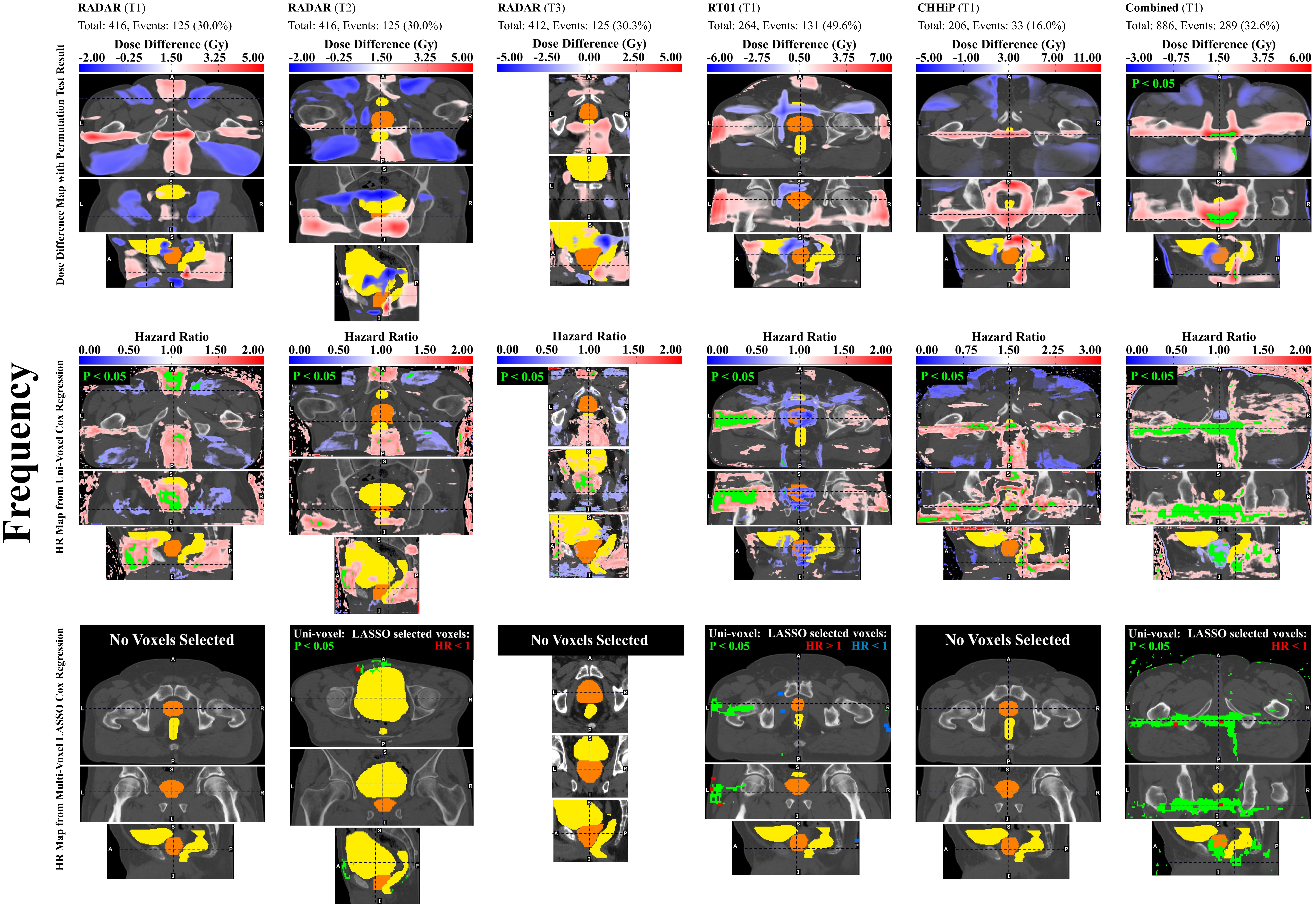

RADAR (T1)
Total: 416, Events: 125 (30.0%)
RADAR (T2)
Total: 416, Events: 125 (30.0%)
RADAR (T3)
Total: 412, Events: 125 (30.3%)
RT01 (T1)
Total: 264, Events: 131 (49.6%)
CHHiP (T1)
Total: 206, Events: 33 (16.0%)
Combined (T1)
Total: 886, Events: 289 (32.6%)
Frequency
Dose Difference Map with Permutation Test Result
Dose Difference (Gy)
-2.00 -0.25 1.50 3.25 5.00
-2.00 -0.25 1.50 3.25 5.00
-5.00 -2.50 0.00 2.50 5.00
-6.00 -2.75 0.50 3.75 7.00
-5.00 -1.00 3.00 7.00 11.00
-3.00 -0.75 1.50 3.75 6.00
P < 0.05
HR Map from Uni-Voxel Cox Regression
Hazard Ratio
0.00 0.50 1.00 1.50 2.00
0.00 0.75 1.50 2.25 3.00
P < 0.05
HR Map from Multi-Voxel LASSO Cox Regression
No Voxels Selected
Uni-voxel: LASSO selected voxels:
P < 0.05
HR < 1
HR > 1

*Section 4: Mean and Standard Deviation Dose Distributions*

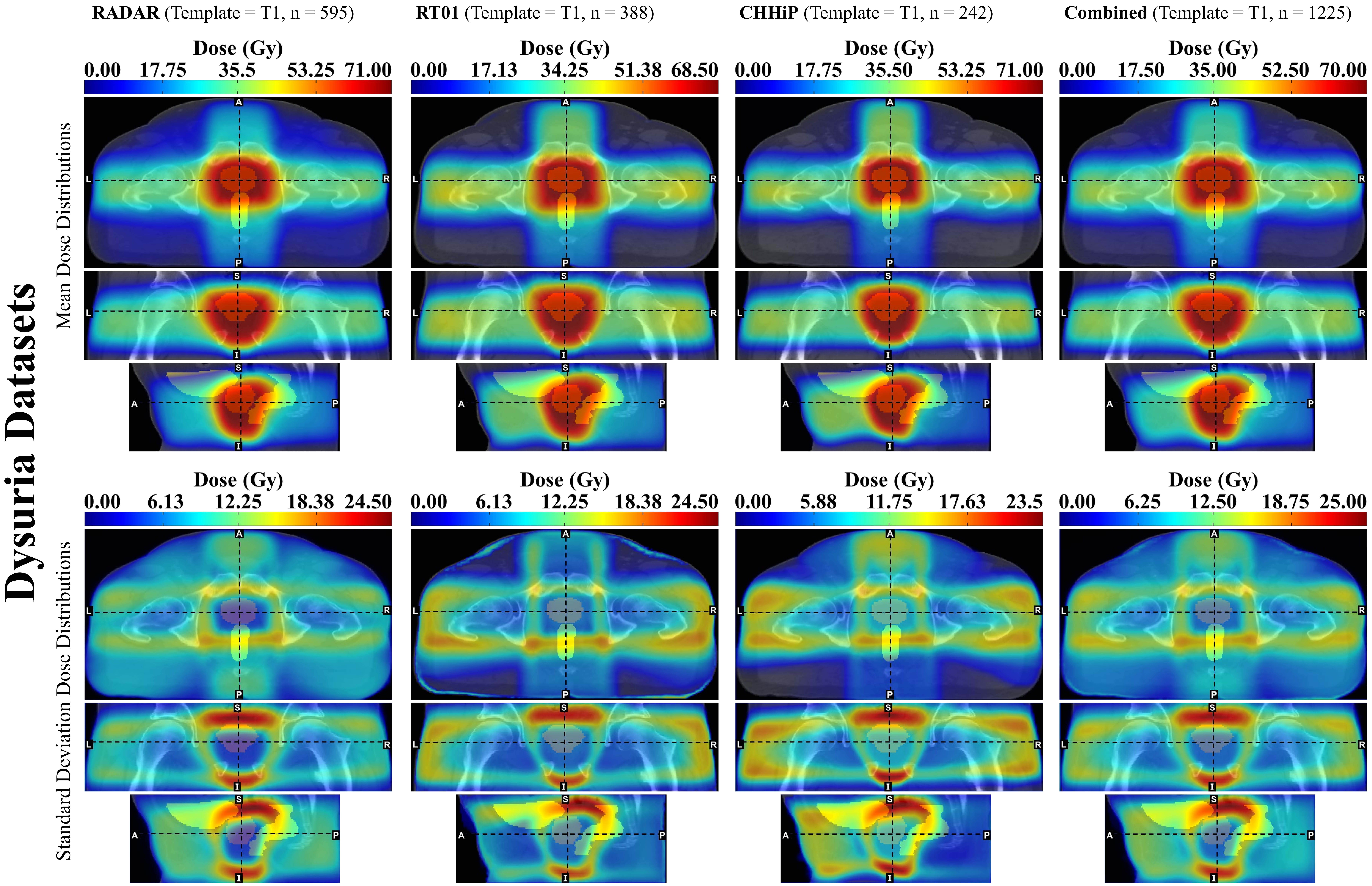

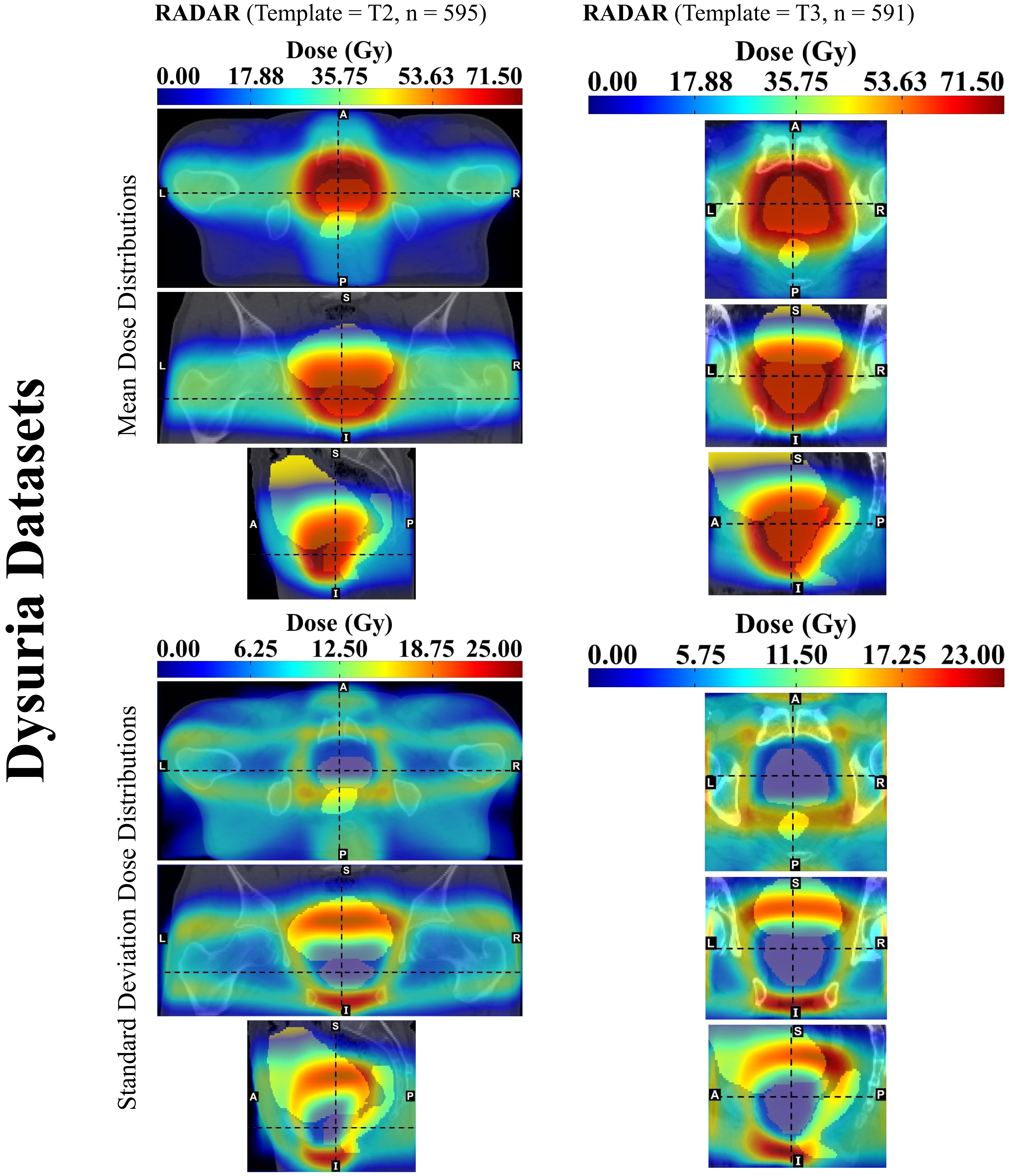

Dysuria Datasets
RADAR (Template = T2, n = 595)
RADAR (Template = T3, n = 591)
Mean Dose Distributions
Dose (Gy)
0.00 17.88 35.75 53.63 71.50
Dose (Gy)
0.00 17.88 35.75 53.63 71.50
Standard Deviation Dose Distributions
Dose (Gy)
0.00 6.25 12.50 18.75 25.00
Dose (Gy)
0.00 5.75 11.50 17.25 23.00

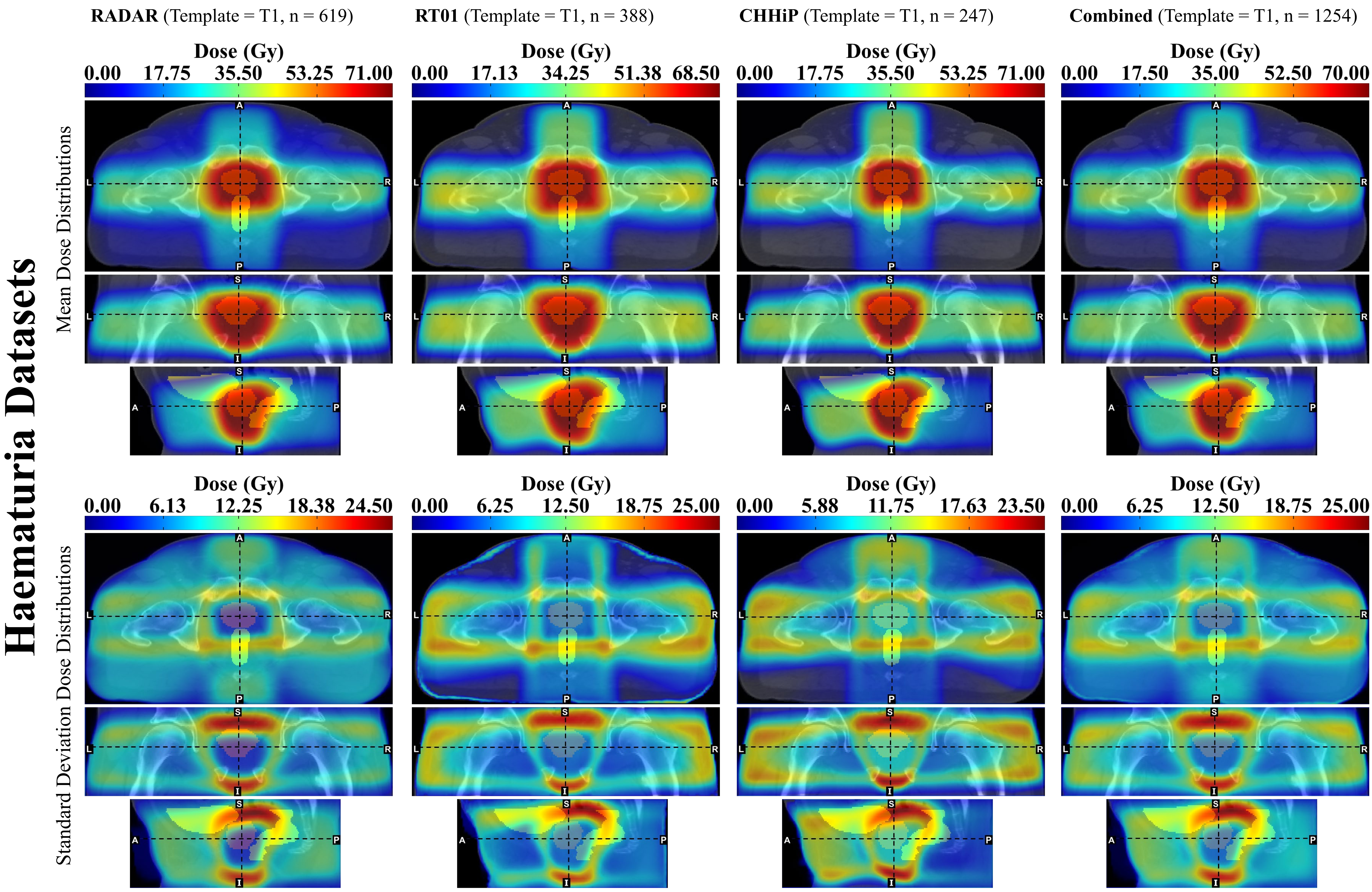

Haematuria Datasets
Mean Dose Distributions
Standard Deviation Dose Distributions
**RADAR** (Template = T1, n = 619)
**RT01** (Template = T1, n = 388)
**CHHiP** (Template = T1, n = 247)
**Combined** (Template = T1, n = 1254)
Dose (Gy)
0.00 17.75 35.50 53.25 71.00
0.00 17.13 34.25 51.38 68.50
0.00 17.75 35.50 53.25 71.00
0.00 17.50 35.00 52.50 70.00
0.00 6.13 12.25 18.38 24.50
0.00 6.25 12.50 18.75 25.00
0.00 5.88 11.75 17.63 23.50
0.00 6.25 12.50 18.75 25.00

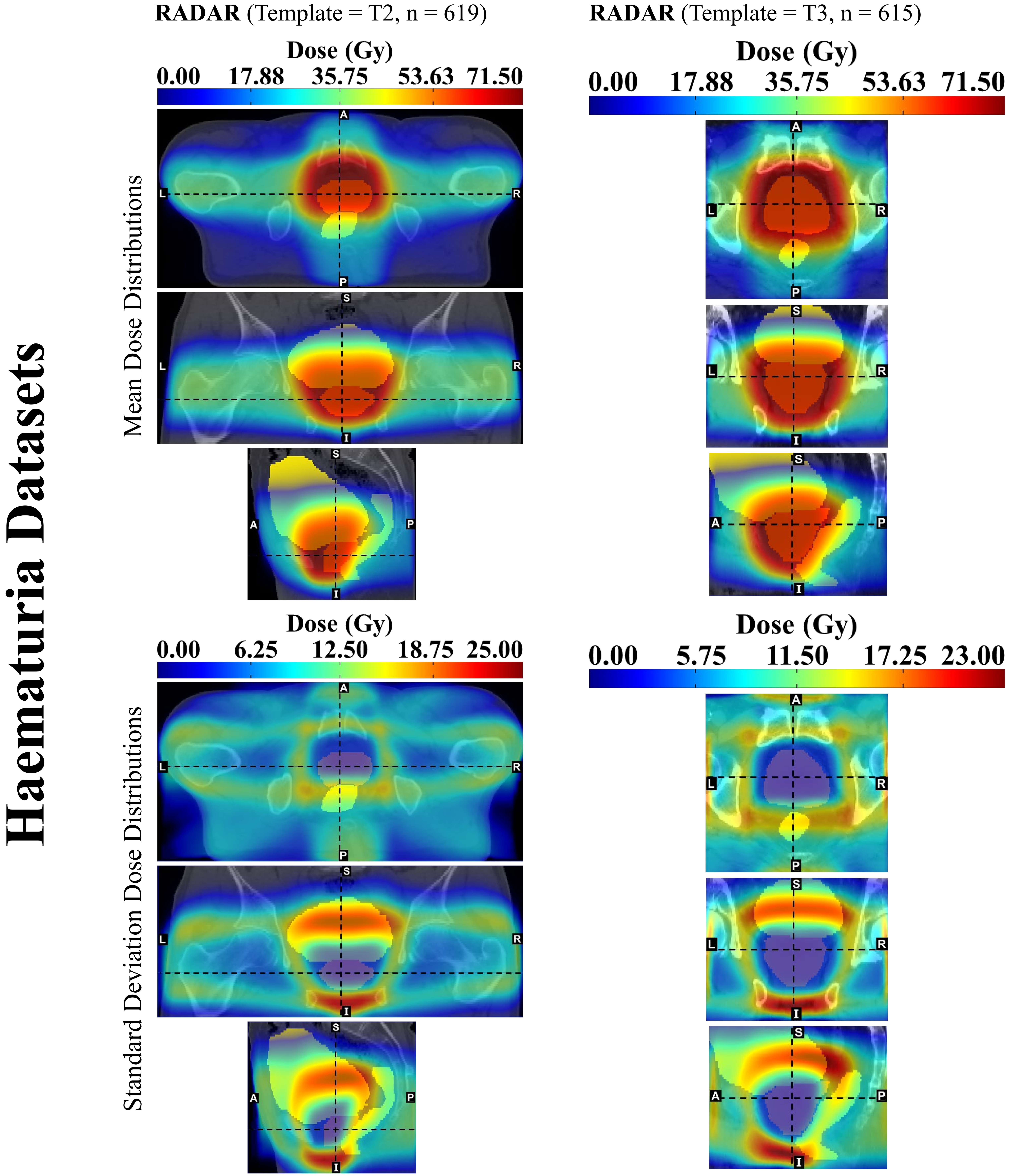

Haematuria Datasets
RADAR (Template = T2, n = 619)
RADAR (Template = T3, n = 615)
Mean Dose Distributions
Standard Deviation Dose Distributions
Dose (Gy)
0.00 17.88 35.75 53.63 71.50
0.00 6.25 12.50 18.75 25.00
0.00 5.75 11.50 17.25 23.00
A
P
L
R
S
I

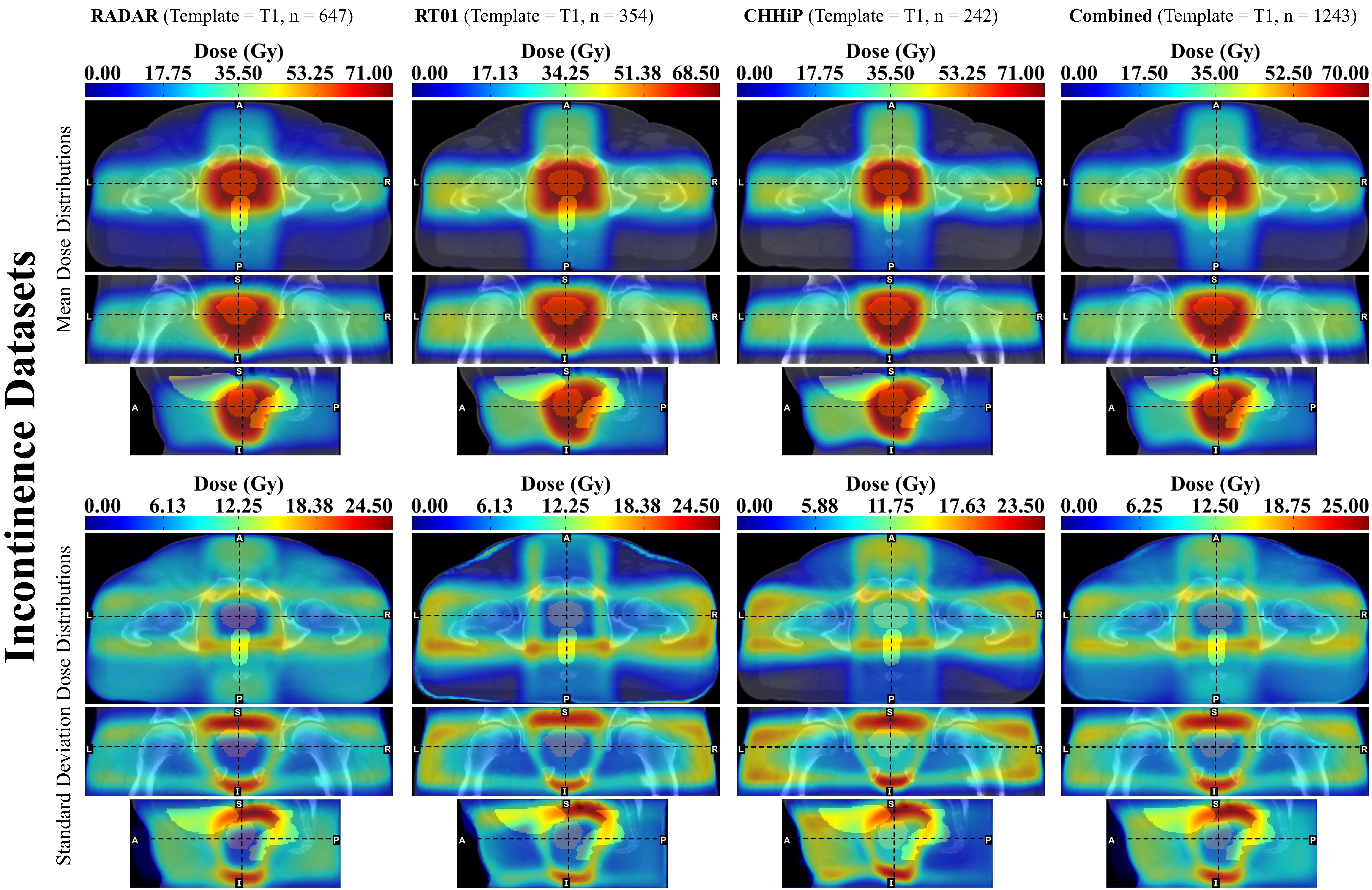

Incontinence Datasets
Mean Dose Distributions
Standard Deviation Dose Distributions
RADAR (Template = T1, n = 647)
RT01 (Template = T1, n = 354)
CHHiP (Template = T1, n = 242)
Combined (Template = T1, n = 1243)
Dose (Gy)
0.00 17.75 35.50 53.25 71.00
0.00 17.13 34.25 51.38 68.50
0.00 17.75 35.50 53.25 71.00
0.00 17.50 35.00 52.50 70.00
0.00 6.13 12.25 18.38 24.50
0.00 6.13 12.25 18.38 24.50
0.00 5.88 11.75 17.63 23.50
0.00 6.25 12.50 18.75 25.00

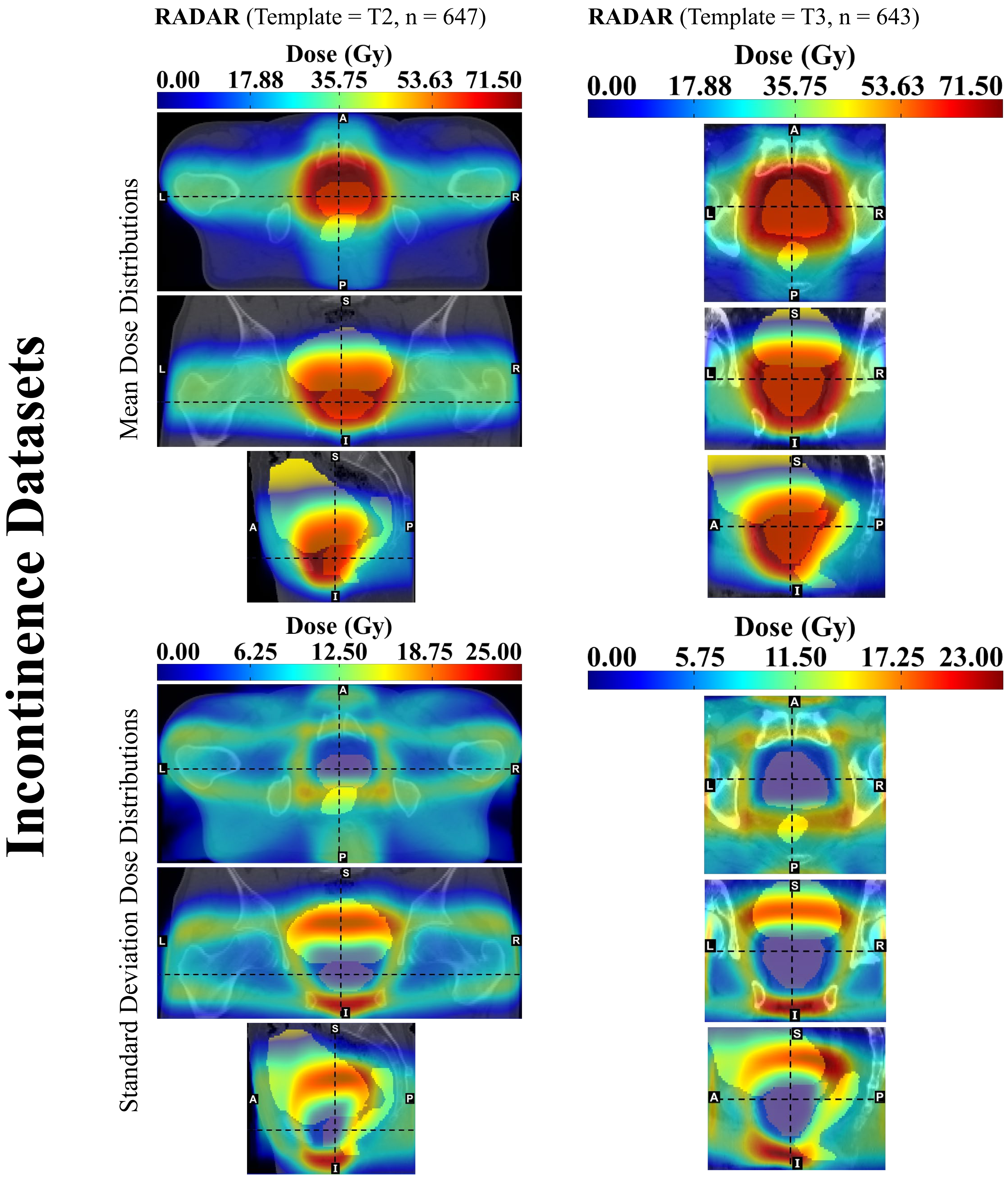

Incontinence Datasets
RADAR (Template = T2, n = 647)
RADAR (Template = T3, n = 643)
Mean Dose Distributions
Standard Deviation Dose Distributions
Dose (Gy)
0.00 17.88 35.75 53.63 71.50
Dose (Gy)
0.00 17.88 35.75 53.63 71.50
Dose (Gy)
0.00 6.25 12.50 18.75 25.00
Dose (Gy)
0.00 5.75 11.50 17.25 23.00
A
P
L
R
S
I

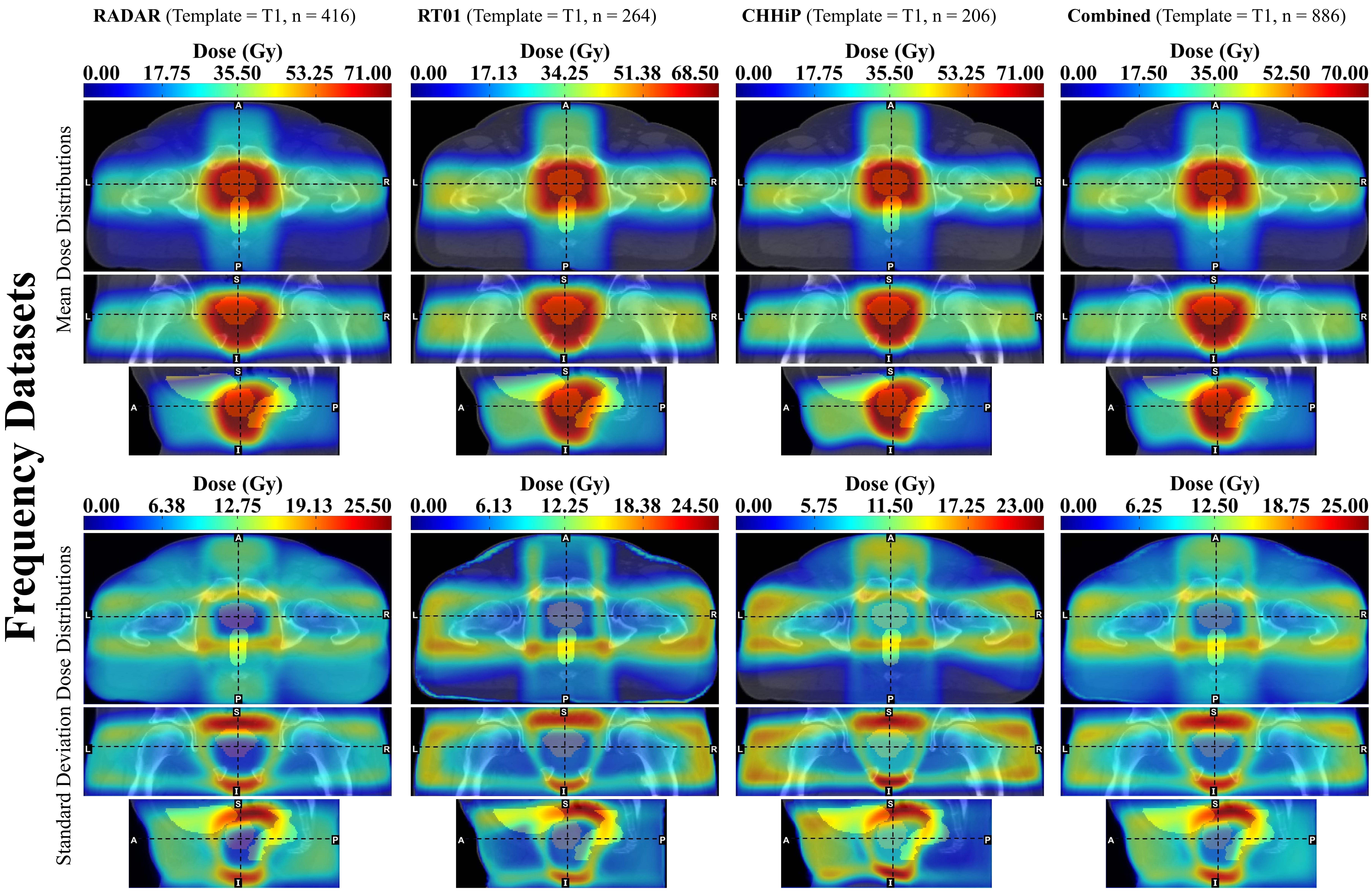
Frequency Datasets
RADAR (Template = T1, n = 416)
RT01 (Template = T1, n = 264)
CHHiP (Template = T1, n = 206)
Combined (Template = T1, n = 886)
Mean Dose Distributions
Standard Deviation Dose Distributions
Dose (Gy)
0.00 17.75 35.50 53.25 71.00
0.00 17.13 34.25 51.38 68.50
0.00 17.75 35.50 53.25 71.00
0.00 17.50 35.00 52.50 70.00
0.00 6.38 12.75 19.13 25.50
0.00 6.13 12.25 18.38 24.50
0.00 5.75 11.50 17.25 23.00
0.00 6.25 12.50 18.75 25.00
A
P
L
R
S
I

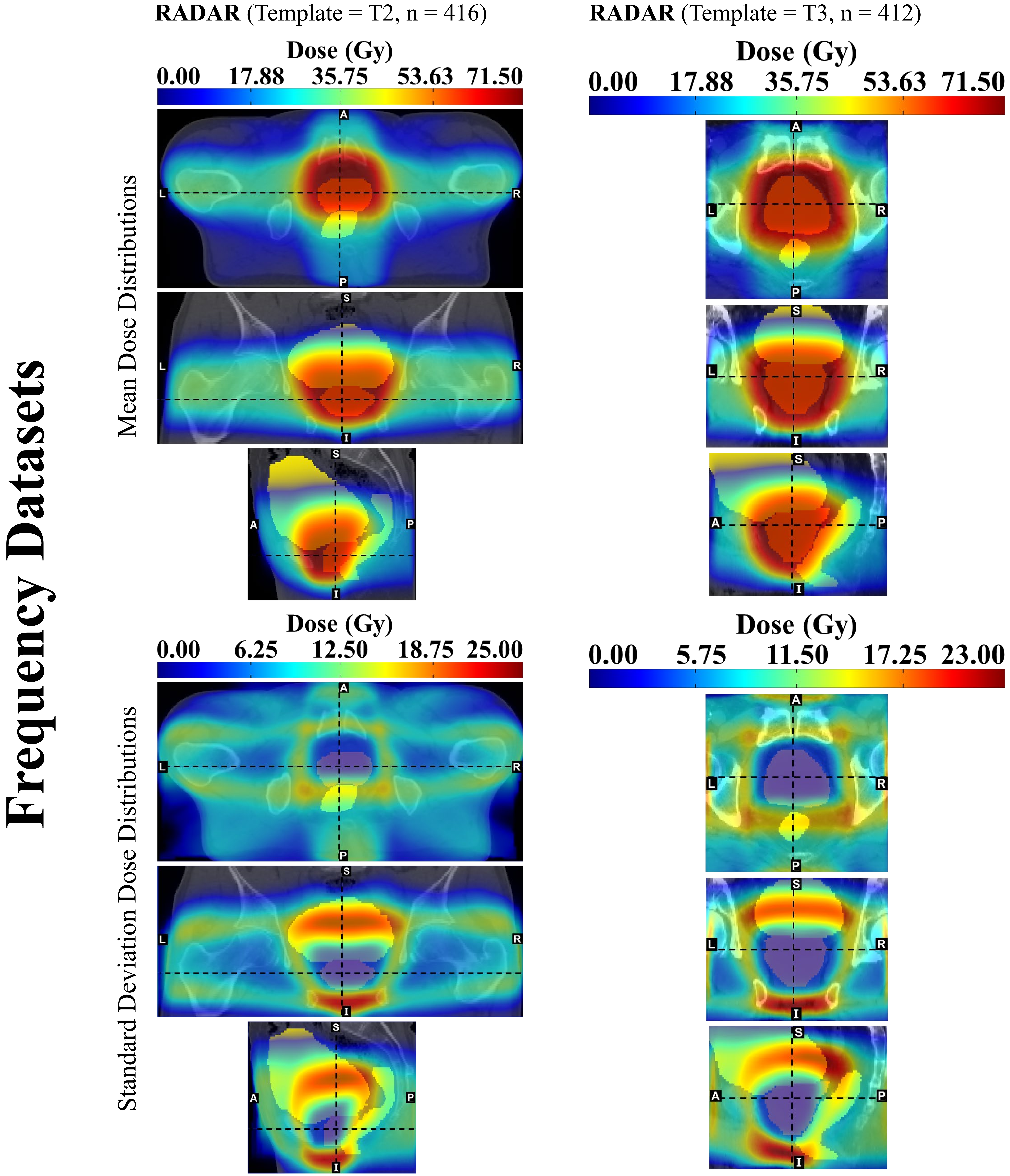

Frequency Datasets
RADAR (Template = T2, n = 416)
RADAR (Template = T3, n = 412)
Mean Dose Distributions
Standard Deviation Dose Distributions
Dose (Gy)
0.00 17.88 35.75 53.63 71.50
Dose (Gy)
0.00 17.88 35.75 53.63 71.50
Dose (Gy)
0.00 6.25 12.50 18.75 25.00
Dose (Gy)
0.00 5.75 11.50 17.25 23.00

***References***


1. Peduz P, Concato J, Kemper E, Holford TR, Feinstein AR. Study of the Number of Events per Variable in Logistic Regression Analysis. 1996;49(12):1373–9.
2. Rivest-Hénault D, Dowson N, Greer PB, Fripp J, Dowling JA. Robust inverse-consistent affine CT-MR registration in MRI-assisted and MRI-alone prostate radiation therapy. Med Image Anal. 2015;23(1):56–69.
3. Yoo TS, Ackerman MJ, Lorensen WE, Schroeder W, Chalana V, Aylward S, et al. Engineering and algorithm design for an image processing API: a technical report on ITK-the insight toolkit. Stud Health Technol Inform. 2002;586–92.
4. Vercauteren T, Pennec X, Perchant A, Ayache N. Non-parametric diffeomorphic image registration with the demons algorithm. In: International Conference on Medical Image Computing and Computer-Assisted Intervention. Springer; 2007. p. 319–26.
5. Kennedy A, Dowling JA, Greer PB, Holloway L, Jameson MG, Roach D, et al. Similarity clustering based atlas selection for pelvic CT image segmentation. Med Phys. 2019;46(5):2246–50.
6. Smith SM, Nichols TE. Threshold-free cluster enhancement: Addressing problems of smoothing, threshold dependence and localisation in cluster inference. Neuroimage. 2009;44(1):83–98.
